# Transcriptomic-guided whole-slide image classification for molecular subtype identification

**DOI:** 10.1371/journal.pcbi.1013950

**Published:** 2026-02-09

**Authors:** Weiwen Wang, Xiwen Zhang, Yuanyan Xiong

**Affiliations:** 1 Department of Mathematics, College of Information Science and Technology, Jinan University, Guangzhou, Guangdong, China; 2 Department of Bioinformatics, College of Medical Information Engineering, Guangdong Pharmaceutical University, Guangzhou, Guangdong, China; 3 Department of Biochemistry, Key Laboratory of Gene Engineering of the Ministry of Education, School of Life Sciences, Sun Yat-sen University, Guangzhou, Guangdong, China; University of Zurich Faculty of Mathematics and Science: Universitat Zurich Mathematisch-Naturwissenschaftliche Fakultat, SWITZERLAND

## Abstract

Recent advancements in computational pathology have greatly improved automated histopathological analysis. A compelling question in the field is how morphological traits are associated with genetic characteristics or molecular phenotypes. Here we propose TEMI, a novel framework for molecular subtype classification of cancers using whole-slide images (WSIs), augmented with transcriptomic data during training. TEMI aims to extract molecular-level signals from WSIs and make efficient use of available multimodal data. To this end, TEMI introduces a patch fusion network that captures dependencies among local patches of gigapixel WSIs to produce global representations and aligns them with transcriptomic embeddings attained from a masked transcriptomic autoencoder. TEMI achieves superior performance compared with existing methods in molecular subtype classification, owing to its effective integration of transcriptomic information achieved by the two developed alignment strategies. Guided by discriminative transcriptomic data, TEMI learns invariant WSI representations, while morphological features also enhance gene expression prediction. These findings suggest that histological features encode latent molecular signals, highlighting the interplay between the tumor microenvironment and cancer transcriptomics. Our study demonstrates how multimodal learning can bridge morphology and molecular biology, providing an effective tool to advance precision medicine.

## Author summary

Cancer’s intrinsic heterogeneity poses significant challenges to effective treatment. Therefore, molecular subtyping that stratifies patients into subgroups based on molecular and genetic distinctions serves as a cornerstone of precision medicine and personalized healthcare. Owing to the strong capability of artificial intelligence (AI) particularly deep learning in discovery of patterns, developing AI models to identify molecular subtypes directly from routinely available haematoxylin–eosin (H&E)-stained histopathology slides can improve the efficiency of patient stratification, enable timely treatment, and reduce medical costs. A major challenge lies in uncovering how morphological features revealed in H&E-stained slides relate to variation in molecular signals, bridging the gap between phenotypes at different layers of the biological hierarchy. To address this issue, we proposed TEMI, a method for inferring molecular subtypes from H&E-stained histopathology slides, in which the discriminative representation learning of morphological characteristics is guided by transcriptomic profiles. The experiments showed that the proposed method achieved superior performance compared with existing tools, and the guidance of transcriptomic profiles helped to learn stable representations of morphological features in change of imaging techniques. Moreover, morphological features were shown to benefit gene expression prediction. These results suggest that the developed tool is effective for molecular subtype identification from histopathology slides and underscore the links between cancer morphology and transcriptomics.

## Introduction

Histopathological images depict morphological and architectural details consisted of thousands of cells from tumor tissue sections in situ, and have been routinely used for cancer diagnostics. Computational pathology, which leverages computational methods for histopathological image analysis, has advanced rapidly with the rise of machine learning, particularly deep learning [1]. Deep learning methods have shown their strong capabilities of learning imperceptible patterns from whole-slide images (WSIs) of tumor sections for various downstream tasks, including classification of cancer types [2,3], prediction of gene mutation [4,5], and survival analysis [6,7].

Particularly, predicting molecular subtypes of cancers from histopathological images using deep learning has become a major area of interest, as it may accelerate diagnosis and enable more precise and timely clinical care. For example, Kather et al. showed that a ResNet18 model can detect microsatellite instability (MSI)—a phenotype arising from deficiencies in the DNA mismatch repair (MMR) system—at a satisfactory level in gastrointestinal cancers from WSIs [8]. Their study also estimated that deep-learning–based MSI screening could substantially reduce medical costs compared with the standard immunohistochemistry workflow. More recently, Saillard et al developed MSIntuit, a clinically approved deep-learning based pre-screening tool for MSI detection from haematoxylin-eosin (H&E)-stained slides [9]. In a related study, Chang et al. proposed a self-attention-based convolutional neural network for MSI classification in colorectal adenocarcinoma using a multicenter Chinese cohort [10]. In [11] and [12], researchers developed a clinically applicable ResNet18 model to distinguish four multi-omics integrative subtypes of hormone receptor-positive (HR ^+^)/human epidermal growth factor receptor 2-negative (HER2^−^) breast cancer and demonstrated the superiority of subtyping-directed precision treatment strategies.

A significant challenge in applying deep learning methods to digital pathology lies in learning a compact representation of giga-pixel-sized histopathological images. Traditionally, regions of interest are selected by experienced pathologists, and then convolutional neural networks are trained by these well-curated data [7,13]. This approach is highly dependent on annotated labels and is labor-intensive. Semi-supervised learning methods aim to address this issue by leveraging limited local annotations more efficiently [14–16]. Meanwhile, multiple instance learning methods offer a more comprehensive solution [17] by capturing dependencies among local patches within a WSI through various attention mechanisms [18–21], requiring only global annotations. For instance, the pathology foundation model Prov-GigaPath [22] takes a WSI as input and employs the transformer-based LongNet [23] to model the long-range dependencies between local patches. Similarly, recently developed general-purpose foundation models [24–27] are mainly built upon a self-supervised framework DINOv2 [28] with Vision Transformer (ViT) backbones [29] for representation learning of patches and slides. However, these models demand highly expensive computational resources. To mitigate distributional shifts in datasets, self-supervised learning and meta-learning techniques have also been incorporated in learning highly generalized representations [30,31].

Delving into the tumor microenvironment (TME) is crucial for developing potential cancer therapies. Histopathological images that reveal the spatial tissue context of the TME in combination with spatial transcriptomic profiles is a promising forefront to achieve this goal [32,33]. However, the associated costs may limit accessibility for a broad patient population [34]. In this study, we take a step back and focus on integrating histopathological images with bulk transcriptomic data. It has been demonstrated that combining histopathological images with genomic or transcriptomic data significantly improves cancer diagnostics, prognosis, and the identification of biomarkers [6,7,35–37]. Additionally, research has shown that the morphological traits presented in histopathological images are closely associated with gene expression [38,39]. Inspired by these findings, we explore the potential of leveraging WSIs to differentiate molecular subtypes of cancers driven by gene expression changes. This approach could reveal the relationships between TME and molecular subtypes, paving the way for personalized targeted therapies at lower medical costs.

In contrast to previous studies that train models directly on WSIs and may suffer from limited generalization owing to the intricate morphology–molecular relationship [5,8,30], we propose TEMI (**T**ranscriptomic **E**xpression from **M**orphological **I**mages), a method designed to detect molecular subtypes of cancers using WSIs of tumor sections by jointly incorporating morphological features and transcriptomic profiles during training to improve its performance. There are two main challenges: the aforementioned representation learning of giga-pixel images, and the seamless integration of heterogeneous data with significant gaps. We tackle these issues by introducing a patch fusion network and aligning heterogeneous data under inexact conditions. The patch fusion network employs a multi-head dot-product attention mechanism to learn low-dimensional representations of WSIs. It automatically weighs patches within an images through their scaled similarities that describe pairwise dependencies, then linear combination of patches follows to yield a global representation. We also build a masked transcriptomic autoencoder to obtain compact representations of transcriptome in a low-dimensional space via self-supervised learning. To exploit the discriminative ability of transcriptome, we align paired data from two sources in a shared low-dimensional space by orthogonal decomposition of representations of transcriptomic data with the error term to capture the unnecessary parts to achieve inexact alignment, aiming to mitigate the disparities between sources. We also establish partial alignment between representations by using representations of WSIs for masked transcriptomic data reconstruction.

To evaluate the capability of TEMI and investigate the relationship between morphological features and transcriptomic profiles, we conducted a comprehensive analysis across three TCGA cancer cohorts: colorectal cancer (CRC; including colon and rectal adenocarcinomas), stomach adenocarcinoma (STAD), and glioblastoma multiforme (GBM), in which TEMI showed superior performance compared with existing approaches.

## Results

### Overview of TEMI

**Fig 1:**
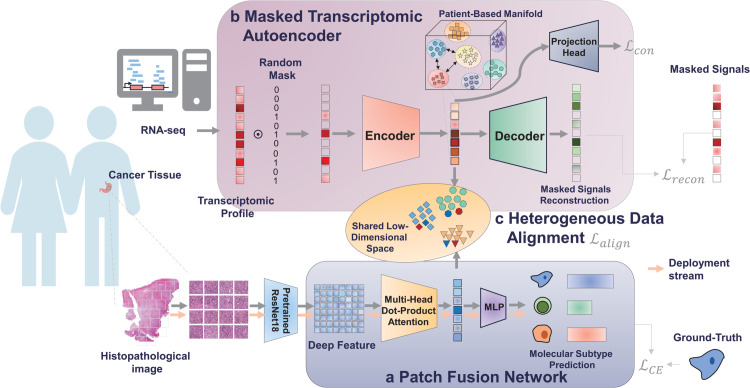
Overview of TEMI. **a** Patch fusion network generates representations of whole-slide images (WSIs) through a stacked multi-head dot-product attention. First, deep features of tiled patches are exacted by a pretrained convolutional neural network. Then the stacked multi-head dot-product attention integrates a collection of deep features into a low-dimensional representation by adaptively weighing deep features and patches. Predictions are made by a multi-layer perceptron (MLP). **b** Masked transcriptomic autoencoder reconstructs masked transcriptomic signals from the expression of unmasked genes, implicitly revealing the underlying relationships between genes. Using contrastive learning, the masked transcriptomic data are projected into a patient-based manifold, where compact, patient-oriented groups emerge, facilitating structured representations. **c** Heterogeneous data alignment maps the representations of WSIs and transcriptomic data into a shared low-dimensional space via orthogonal decomposition and partial reconstruction. This process injects discriminative information from transcriptomic signals into the patch fusion network while addressing the inherent modality gap between WSIs and transcriptomic data through inexact alignment. During deployment, only WSIs are required for molecular subtype prediction.

Our method comprises three key components: (1) a patch fusion network, (2) a masked transcriptomic autoencoder (MTA), and (3) heterogeneous data alignment. In our framework, WSIs are divided into non-overlapping patches, and deep features from these patches are extracted using a pretrained convolutional neural network. The patch fusion network integrates the deep features of patches from the same patient into a low-dimensional representation by a stacked multi-head dot-product attention, which automatically weighs the importance of deep features and patches. A multi-layer perceptron follows for decision. The architecture of TEMI is presented in Fig 1.

The MTA learns latent representations of bulk transcriptomic data through a masked autoencoder framework [40]. Specifically, it reconstructs expression signals of masked genes using information from unmasked genes aiming to capture the underlying relationships between genes in a low-dimensional space. Due to the randomness of masking, multiple masked inputs can be generated for each patient. To ensure compact and meaningful representations, contrastive learning [41,42] is employed so that data generated from the same patient are mapped closely together, while those from different patients are well-separated.

To incorporate discriminative information from transcriptomic data, we propose heterogeneous data alignment. The representations of WSIs and transcriptomic data are projected into a shared low-dimensional space, ensuring that corresponding features from both modalities are closely aligned. However, exact alignment may not always be achievable due to the inherent gap between WSIs and molecular expression signals. To address this issue, we introduce orthogonal decomposition and partial reconstruction for inexact alignment, denoted by AOR (**A**lignment by **O**rthogonal **D**ecomposition) and APR (**A**lignment by **P**artial **R**econstruction), respectively, which allows us to separate modality-specific components while still aligning shared information across the two modalities. In deployment, only patch fusion network is employed from molecular subtype classification.

### Molecular subtype classification

We verified our method using three cancer cohorts from TCGA—colorectal cancer, stomach adenocarcinoma, and glioblastoma multiforme—to identify molecular cancer subtypes based on formalin-fixed paraffin-embedded (FFPE) H&E-stained histopathology slides, and for each sample, bulk RNA-seq gene-expression data served as its transcriptomic profile. These cohorts are referred to as CRC-DX, STAD-DX, and GBM-DX, respectively. In both CRC-DX and STAD-DX, we aimed to distinguish microsatellite instable (MSI) from microsatellite stable (MSS) subtypes, whereas in GBM-DX, the goal was to classify tumors into the Proneural (Pron.) and Mesenchymal (Mese.) subtypes (see the Data and preprocessing section for details of datasets). We compared our method with two groups of approaches: (1) majority-voting based methods, where predictions are made using local patches of slides and the final decision is obtained through majority voting, and (2) one-slide-as-a-whole based methods, where low-dimensional representations of the entire slide are learned using a patch fusion method and used for prediction by a classifier. The first group includes ResNet18 [8] and MetaCon [30], while the second group includes ABMIL (Attention-based Multiple Instance Learning) [43] and 1Dconv (Convolutional One-Dimensional Layers) [44]. We also compared with TEMI trained without transcriptomic data indicated by TEMI w/o G. The models ABMIL and 1Dconv trained with transcriptomic data are denoted by ABMIL w/G and 1Dconv w/G, where we employed alignment by orthogonal decomposition (see the Methods section). We evaluated the models using the area under the receiver operating characteristic curve (AUC) calculated at the patient level. The median and 95% confidence intervals from 1000-fold bootstrapping for test samples are presented Table 1. Three of TEMI variants are indicated by TEMI w/AOD, TEMI w/APR, and TMEI w/AOD+APR (see the Methods section for details of these variants).

**Table 1:** Performance of compared methods in three cancer cohorts. We use AUC as evaluation metric. The median and 95% confidence intervals (in parentheses) from 1000-fold bootstrapping for test samples are reported.

Methods	CRC-DX MSI vs. MSS	STAD-DX MSI vs. MSS	GBM-DX Pron. vs. Mese.
ResNet18	0.7513	0.8107	0.7390
	(0.6169-0.8727)	(0.6898-0.9061)	(0.7242-0.7532)
MetaCon	0.9008	0.7291	–
	(0.8101-0.9599)	(0.5892-0.8446)	
1Dconv	0.8924	0.8322	0.7325
	(0.7980-0.9546)	(0.7326-0.9101)	(0.5827-0.8544)
ABMIL	0.8551	0.8141	0.7097
	(0.7470-0.9386)	(0.7101-0.8935)	(0.5657-0.8374)
TEMI w/o G	0.9043	0.8251	0.7755
	(0.8259-0.9608)	(0.7243-0.9076)	(0.6467-0.8913)
1Dconv w/G	0.8944	**0.8424**	0.7401
	(0.7971-0.9643)	(0.7491-0.9122)	(0.5938-0.8600)
ABMIL w/G	0.8862	0.8386	0.7333
	(0.7833-0.9582)	(0.7414-0.9156)	(0.5995-0.8581)
TEMI w/AOD	0.9148	0.8304	**0.8003**
	(0.8377-0.9661)	(0.7278-0.9089)	(0.6653-0.9174)
TEMI w/APR	0.9116	0.8181	0.7728
	(0.8412-0.9634)	(0.7202-0.8930)	(0.6353-0.8813)
TMEI w/AOD+APR	**0.9232**	0.8322	0.7893
	(0.8624-0.9720)	(0.7319-0.9022)	(0.6547-0.9019)

In CRC-DX, TMEI w/AOD+APR achieved the best median AUC (92.32%), while 1Dconv w/G outperformed the rest with the median AUC 84.24% in STAD-DX. In GBM-DX, TEMI w/AOD had the best median AUC 80.03%. Overall, variants of TEMI show comparative performance in the three cancer cohorts. For TEMI, ABMIL, and 1Dconv, incorporating transcriptomic profiles during training yields incremental gains for slide-based molecular subtype classification.

Compared to other methods without transcriptomic integration, TEMI w/o G consistently achieved superior performance across all three cancer cohorts, indicating the effectiveness of the proposed patch fusion network in capturing global representations of WSIs.

### Transferable representation learning of different sources.

To assess whether transcriptomic data contributes to learning transferable features, we trained variants of TEMI using transcriptomic profiles and FFPE slides from the CRC-DX dataset and evaluated them on the CRC-KR dataset, which consists of snap-frozen colorectal cancer samples, for MSI vs. MSS classification.

Compared to TEMI trained without transcriptomic data, variants of TEMI showed improved performance when transcriptomic profiles were incorporated. We also compared our models with ResNet18 and MetaCon, which were directly trained and evaluated on the CRC-KR dataset. TMEI w/AOD+APR achieved the best AUC of 80.04% in the transfer learning setting, close to ResNet18 (82.15%) and MetaCon (84.09%). Table 2 summarizes these results, demonstrating that transcriptomic data enhances transfer learning in our models.

**Table 2:** Evaluation on transfer learning. Variants of TEMI trained FFPE slides were evaluated by snap-frozen colorectal cancer samples. The median and 95% confidence intervals (CI) of AUC from 1000-fold bootstrapping for test samples are reported.

Learning paradigms	Methods		AUC	95% CI
Supervised learning	ResNet18		0.8215	0.7246-0.9109
	MetaCon		**0.8409**	0.7475-0.9193
Transfer learning	TEMI	w/o G	0.7638	0.6392-0.8642
		w/AOD	0.7690	0.6464-0.8685
		w/APR	0.7757	0.6549-0.8706
		w/AOD+APR	**0.8004**	0.6927-0.8945

**Fig 2:**
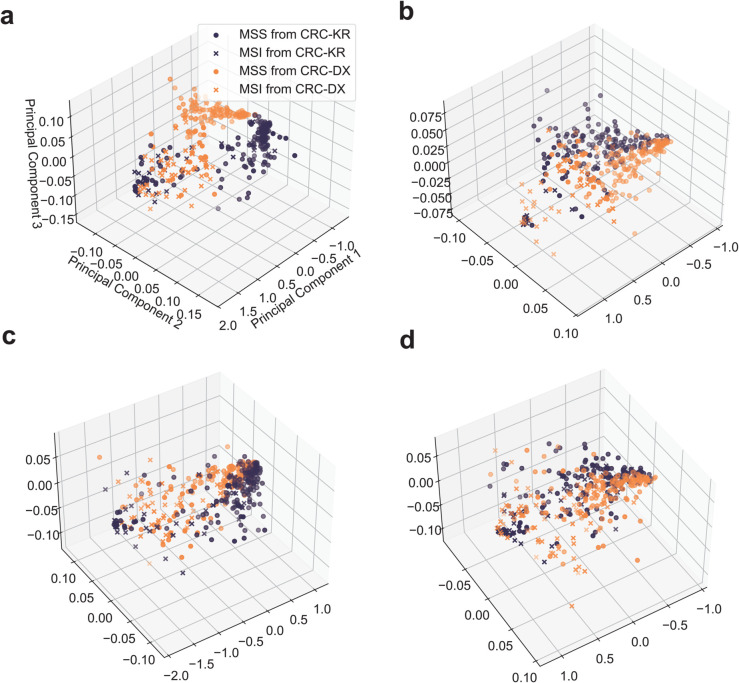
Visualization of low-dimensional representations of snap-frozen and FFPE samples from colorectal cancer cohort. **a** TEMI trained using whole-slide images only (TEMI w/o G). **b** TEMI incorporating alignment by partial reconstruction (TEMI w/APR). **c** TEMI incorporating alignment by orthogonal decomposition (TEMI w AOD). **d** TEMI combining both AOD and APR modules (TEMI w/AOD+APR).

For each variant of TEMI, we extracted the representations of WSIs from the test samples of CRC-DX and CRC-KR. Principal component analysis was employed to project the representations into a three-dimensional space, thereby reducing the impact of high dimensionality on the distance metric. Fig 2 displays 200 randomly selected samples from each subtype. For each subtype, we then computed the pairwise cosine distance between the representations from the two sources. The average cosine distance, normalized by the average pairwise cosine distance of the same subtype within the CRC-KR dataset, is reported in Table 3. For all variants of TEMI, the normalized average pairwise inter-source distance was less than 1, indicating that representations of samples from CRC-DX and CRC-KR are more similar to each other than representations of samples within CRC-KR. This may explain why all TEMI variants achieved fair AUCs in the transfer learning setting. Leveraging transcriptomic profiles generally reduced the gap between representations of snap-frozen and FFPE samples in TEMI, except for TEMI w/APR on the MSI subtype. Overall, TEMI w/AOD+APR benefited the most, consistent with the results shown in Table 2.

**Table 3:** Representation gap between snap-frozen and FFPE samples. The normalized average cosine distance between representations of snap-frozen and FFPE colorectal cancer samples was computed for each subtype. The ΔGap% denotes the relative change in percentage compared to the results obtained from TEMI w/o G, with arrows marking the change direction.

Variants of TEMI	MSI	MSS	Δ Gap%	
w/o G	0.8379	0.9733	–	–
w/AOD	0.8277	0.9308	1.22% ↓	4.37% ↓
w/APR	0.8491	0.9467	1.33% ↑	2.73% ↓
w/AOD+APR	0.8108	0.9414	3.23% ↓	3.27% ↓

The results suggest that integrating WSIs and transcriptomic profiles through co-training enhances the learning of invariant discriminative features for MSI vs. MSS classification in colorectal cancer, contingent on the alignment strategy used.

### Contribution of morphological features to gene expression prediction.

**Fig 3:**
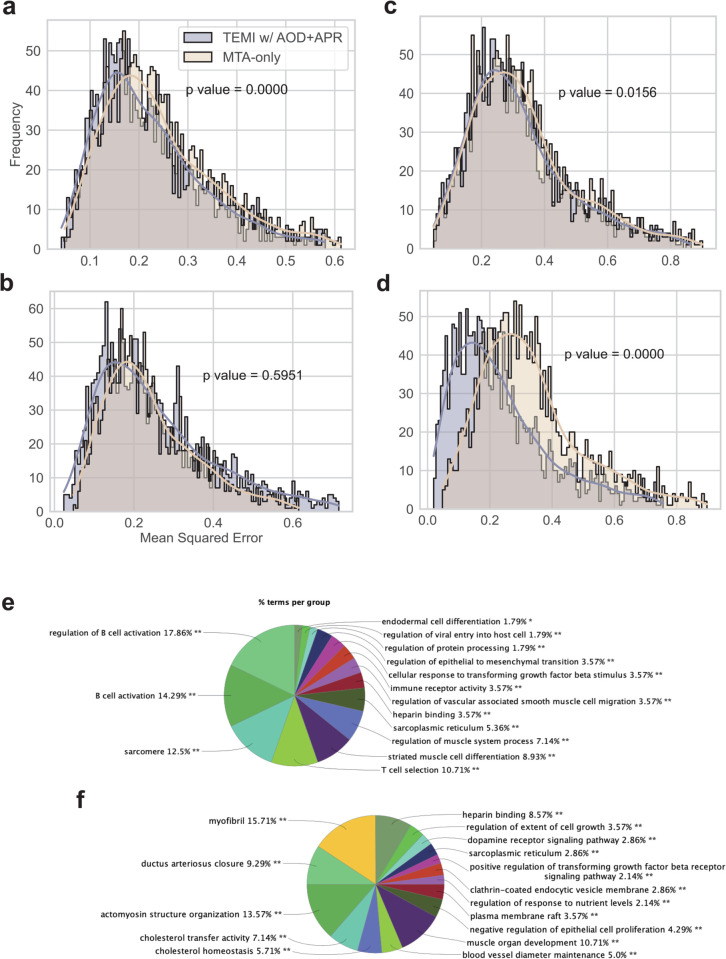
Distributions of mean squared errors (MSEs) of predicted masked genes and pathway enrichment analysis of the top 200 genes with the smallest errors. **a, c** MSS groups of colorectal cancer (CRC) and stomach adenocarcinoma (STAD) samples. **b, d** MSI groups of CRC and STAD samples. **e, f** Enriched pathways of the top 200 low-error genes in the MSS and MSI subtypes of STAD, respectively.

To investigate the connections between morphological features and transcriptomic profiles, we evaluated MTA and TMEI w/AOD+APR for masked gene expression prediction and reported their mean squared errors (MSEs) on CRC and STAD test samples. Distributions of errors are shown in Fig 3a–3d. A one-sided Mann-Whitney *U*-test was conducted to determine whether MSEs of TMEI w/AOD+APR were significantly lower than that of MTA. The results suggest morphological features benefit gene expression prediction for MTA, except in the MSI subtype of CRC samples. It provides evidence of connections between the morphological characteristics of tissues and their molecular profiles.

We performed gene ontology (GO) enrichment analysis using ClueGO (version 2.5.10) for the top 200 genes with the smallest MSEs in MSS and MSI subtypes of STAD samples, respectively. A Benjamini–Hochberg corrected *p*-value threshold of ≤ 0.05 was applied, GO terms were required to contain at least three genes(≥ 3). The enriched GO terms are presented in Fig 3e and 3f (also see Fig A in S1 Appendix). Most of the enriched GO terms are related to cell morphology. For the MSS subtype, the GO terms also involve immune regulation, specifically processes related to immune activation, antibody production, or immune tolerance.

The enrichment reflects genes whose expression levels were better predicted by TMEI w/AOD+APR, which are not necessarily those with higher expression levels and therefore does not conflict with the established knowledge that the MSI subtype is generally characterized by higher immune infiltration and immune activation in stomach adenocarcinoma [45,46]. Although MSI tumors typically exhibit stronger immune activation and infiltration, immune cells in MSS tumors are often sparse and localized, which enhances the morphological contrast between immune and non-immune regions and facilitates model recognition. Consequently, immune-related genes in the MSS subtype may exhibit lower prediction errors, reflecting the model’s ability to capture morphologically distinct immune features rather than indicating stronger overall immune activity.

### Ablation study on alignment strategies.

To validate the effectiveness of our heterogeneous data alignment methods—alignment by orthogonal decomposition (AOD), alignment by partial reconstruction (APR), and their combination (AOD + APR)—we compared various approaches for integrating transcriptomic data and WSIs. The methods compared include: (i) Mean Squared Error (MSE); (ii) Similarity Consistency (SimC); (iii) Hilbert-Schmidt Independence Criterion (HSIC); (iv) Maximum Mean Discrepancy (MMD); and (v) Gaussian Wasserstein Distance (GWD). These methods are commonly employed in multi-modal learning and transfer learning for data alignment (see the Methods section for details).

Results are presented in Table 4. In CRC-DX, MSE achieved the highest AUC, while in STAD-DX AOD + APR performed best, and in GBM-DX MMD yielded the highest score. Although no single alignment method can be regarded as universally dominant, the consistent top-3 performance of AOD + APR across all three datasets highlights the effectiveness of the proposed alignment strategies. Notably, AOD consistently outperformed APR, which indicates soft alignment may be preferred for seemingly disparate modalities.

**Table 4:** Comparison of methods for data alignment. We use AUC as evaluation metric. The median and 95% confidence intervals (in parentheses) from 1000-fold bootstrapping for test samples are reported.

Alignment	CRC-DX	STAD-DX	GBM-DX
MSE	**0.9260**	0.8169	0.7844
	(0.856-0.9739)	(0.7193-0.9044)	(0.6350-0.8954)
SimC	0.9182	0.8067	0.7685
	(0.8564-0.9661)	(0.7077-0.8947)	(0.6310-0.8950)
HSIC	0.9168	0.8069	0.7825
	(0.8481-0.9668)	(0.6947-0.8954)	(0.6454-0.8880)
MMD	0.9024	0.8151	**0.8120**
	(0.8233-0.9574)	(0.7019-0.9000)	(0.6787-0.9204)
GWD	0.9174	0.8155	0.7787
	(0.8456-0.9665)	(0.7138-0.9027)	(0.6303-0.8990)
AOD	0.9148	0.8304	0.8003
	(0.8377-0.9661)	(0.7278-0.9089)	(0.6653-0.9174)
APR	0.9116	0.8181	0.7728
	(0.8412-0.9634)	(0.7202-0.8930)	(0.6353-0.8813)
AOD+APR	0.9232	**0.8322**	0.7893
	(0.8624-0.9720)	(0.7319-0.9022)	(0.6547-0.9019)

### Analysis of attention scores

**Fig 4:**
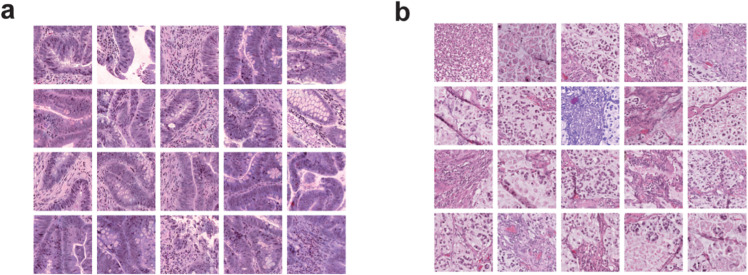
Top 20 patches ranked by attention scores of two samples from CRC-DX. **a** TCGA-AA-3837 from the MSS group. **b** TCGA-WS-AB45 from the MSI group.

Interpretability is of great concern for computational models applied to histopathological images, as it provides users with supporting evidence for the model’s decisions. We demonstrated the interpretability of TEMI using patch-level attention scores on the CRC-DX cohort, with computational details provided in the Methods section. Two test samples were randomly selected: TCGA-AA-3837 from the MSS tumor and TCGA-WS-AB45 from the MSI tumor. Top 20 patches of the two samples with the highest attention scores are presented in Fig 4. Additional results, including the top 20 patches ranked by attention scores from CRC-DX, STAD-DX, and GBM-DX test samples, are shown in Figs B-D in S1 Appendix. The results demonstrate clear morphological distinctions between the molecular subtypes and consistent characteristics within each group. Heatmaps of patch-level attention scores from four GBM-DX test samples are also presented in Fig E in S1 Appendix.

To further explore the effectiveness of the selected patches, we treat patches demonstrated in Fig 4 as templates and analyze their associations with MSS and MSI samples. We define similarity between patches $r_{i}$ and $r_{j}$ by the following formula, where patches are represented by their deep features.


$$sim(r_{i},r_{j})=\exp\left(\frac{r_{i}^{T}r_{j}}{\|r_{i}{\|}_{2}\|r_{j}{\|}_{2}}/\tau \right).$$

In the experiment, the parameter *τ* was set as 0.07.

**Fig 5:**
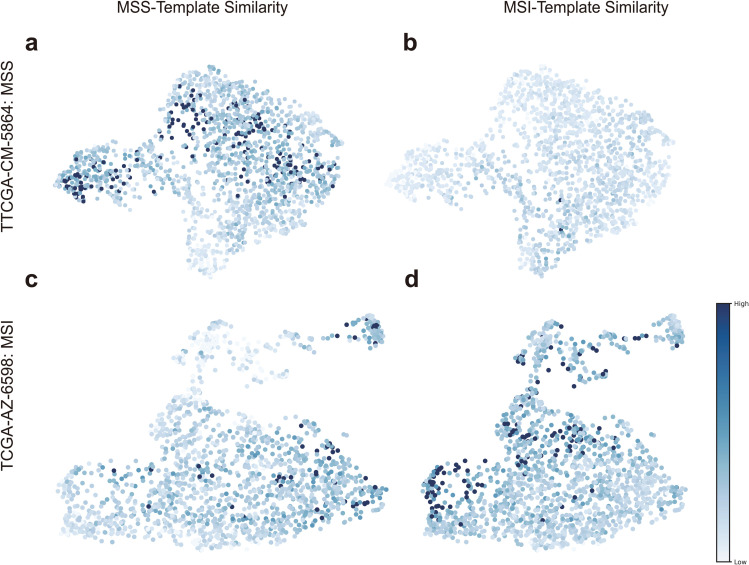
Subtype-specific similarity scores of patches. **a**, **b** The MSS TCGA-CM-5864. **c**, **d** The MSI TCGA-AZ-6598. **a**, **c** Similarity with respect to the MSS templates. **b**, **d** Similarity with respect to the MSI templates.

For each patch in a WSI, its subtype-specific similarity score was computed by averaging its similarity scores between the 20 templates of the respective subtype. We visualized patches of a WSI by UMAP [47] using their deep features, with brightness of each point indicating its subtype-specific similarity. Two test samples, the MSS sample TCGA-CM-5864 and the MSI sample TCGA-AZ-6598, were selected for demonstration. The results are shown in Fig 5. Patches from TCGA-CM-5864 exhibit higher similarity scores with the MSS templates than with the MSI templates, whereas the opposite is observed for TCGA-AZ-6598.

These findings suggest that attention scores may help reveal subtype-relevant morphological characteristics.

### Impact of feature extractors

In our experiments, we employed ResNet18 as the feature extractor for image patches. However, with advanced architectures for visual representation available, such as the ViT, it is natural to question whether ResNet18 is sufficiently effective for TEMI. To address this, we further incorporated DINOv2, a prevailing backbone in foundation models for histopathological image analysis, as a benchmark of comparison. We utilized a pretrained DINOv2 model on ImageNet and subsequently fine-tuned it for tumor detection, where the task was to classify patches as originating from tumor, dense tissue, or loose tissue. The fine-tuning dataset, provided by [8], comprised 11,977 patches, and it was split into 70% for training and 30% for validation. During the fine-tuning stage, only the last ten layers of DINOv2, as well as an additional linear layer for classification, were updated by AdamW with a learning rate of $1\;\times \;10^{-4}$. After 20 epochs, the accuracy reached 99.82% on the training set and 99.64% on the validation set. The fine-tuned model was then used as a feature extractor for TEMI.

As foundation models for computational pathology have emerged in recent years, we also included several of these models as feature extractors for comparison. Specifically, we evaluated: (1) Prov-GigaPath, trained on approximately 170,000 WSIs from the Providence Health Network [22]; (2) H-optimus-1, trained on over 1 million proprietary WSIs [26]; and (3) Virchow, trained on 1.5 million slides from Memorial Sloan Kettering Cancer Center [27]. Additional details of these foundation models are provided in Table A in S1 Appendix.

**Fig 6:**
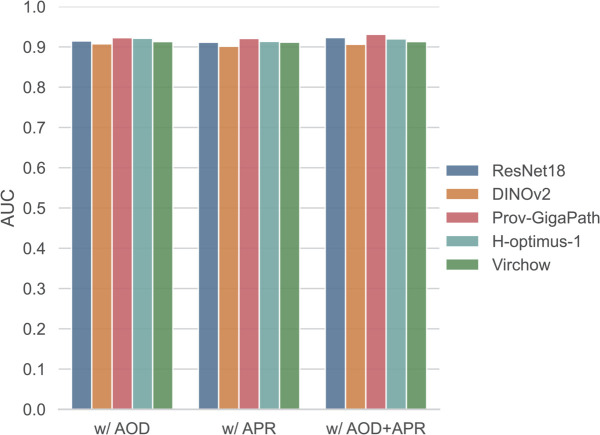
Performance of variants of TEMI on CRC-DX using different feature extractors. Employing the advanced feature extractor does not lead to significant improvement in TEMI.

Fig 6 presents the performance of TEMI variants on the CRC-DX cohort using ResNet18 and the four advanced models as feature extractors. The best median AUC (93.13%) was achieved by TEMI w/AOD+APR using Prov-GigaPath as the feature extractor, followed closely by the variant using ResNet18 (median AUC: 92.32%). The median AUCs achieved by using different feature extractors are summarized in Table B in S1 Appendix. Interestingly, using advanced foundation-model extractors did not yield substantial performance improvements for TEMI. This contrasts with ABMIL, which benefitted considerably from these extractors, with its median AUC increasing from 85.51% to over 90%. For TEMI, the performance differences among simple extractor (ResNet18) and advanced extractors (e.g., Prov-GigaPath) were marginal. This suggests that the architecture of TEMI may already be sufficiently expressive for this task. Meanwhile, these results do not diminish the strong representation-learning capabilities of pathology foundation models, as they require far fewer training epochs to achieve satisfactory performance before overfitting in the downstream classification.

## Discussion

We developed TEMI, a framework for identifying molecular subtypes of cancer from whole-slide images (WSIs), enhanced with transcriptomic data during training. TEMI makes three main contributions. First, it adopts a multimodal-training-with-unimodal-inference paradigm, enabling molecular subtype classification from WSIs while leveraging transcriptomic profiles only at the training stage. Second, to address the challenge of representation learning on giga-pixel WSIs, TEMI develops a patch fusion network that leverages a multi-head dot-product attention mechanism to adaptively weigh features and patches, thereby generating unified representations through linear combinations. For the transcriptomic modality, TEMI employs a masked transcriptomic autoencoder to derive compact representations, where masked reconstruction effectively captures underlying gene relationships. Finally, TEMI presents two alignment strategies that bridge morphological features and molecular signals in a shared low-dimensional space, guiding WSI representations through alignment with discriminative transcriptomic embeddings.

Experiments demonstrate that TEMI achieves superior performance in molecular subtype classification compared with existing methods. In a transfer learning setting, TEMI benefits from the incorporation of transcriptomic data, which guides the learning of invariant representations through heterogeneous data alignment. The proposed alignment strategies—alignment by orthogonal decomposition and alignment by partial reconstruction—prove effective against established alignment approaches. Analysis of attention scores shows that TEMI highlights discriminative local regions associated with different molecular subtypes, supporting the design of the multi-head dot-product attention mechanism. Further, our experiments reveal that morphological features enhance gene expression prediction for the masked transcriptomic autoencoder, confirming associations between WSIs and transcriptomic profiles. TEMI relies on patch-level deep features, and notably, even a relatively simple backbone (ResNet18) is sufficient to yield strong performance. Overall, TEMI provides an effective and efficient framework for molecular subtype classification from WSIs.

This study has several limitations that should be addressed. First, we integrated WSIs only with bulk RNA-seq gene-expression data, whereas additional molecular modalities—such as protein expression and DNA methylation—should be considered in future work. Even advanced foundation-model feature extractors with over a billion parameters trained on large-scale WSIs—such as Prov-GigaPath and H-optimus-1—did not provide substantial improvements over the much simpler feature extractor ResNet18 for TEMI. This suggests that, while foundation models may be necessary for molecular subtype classification due to their ability to achieve satisfactory performance in fewer training epochs before overfitting (see the section *Impact of feature extractors*), they are not sufficient on their own, highlighting that opportunities remain for designing efficient and task-specific architectures in computational pathology.

Although alignment methods are well developed for modalities with clear logical correspondence (e.g., images, audio, and text), aligning WSIs with transcriptomic profiles remains challenging due to the significant modality gap and potentially undiscovered associations. Our results further indicate that discoveries may be highly dependent on the alignment strategies employed; since no single method consistently dominates, future efforts should focus on developing alignment approaches tailored to these modalities.

Another important avenue for future research lies in elucidating the relationship between morphological context and underlying molecular functions or biological processes. Notably, the benefit of morphological features for gene expression prediction was absent in the GBM-DX cohort (see Fig F in S1 Appendix), raising the question of whether such associations are universal across cancers. For the Proneural vs. Mesenchymal classification in the GBM-DX cohort, all methods achieved only fair performance compared with the CRC-DX and STAD-DX cohorts, with the best AUC being 80.03% from TEMI w/AOD. These results suggest that inferring gene expression from H&E morphology is more difficult in GBM than in CRC and STAD. Therefore, the lack of improvement from morphological features is likely due to weaker associations between GBM morphology and its transcriptomic profiles. Moreover, such conclusion may also be affected by model bias as only fair AUC was achieved, and therefore it may require more sophisticated models for representation learning before morphology can meaningfully enhance gene expression prediction.

## Methods

There are two main obstacles in the formulation of our methods: (1) representation learning of giga-pixel-level images, and (2) data alignment of heterogenous data. This section describes how we resolve these challenges.

### Patch fusion network (PFN)

A whole-slide image is tiled into non-overlapped patches. Mathematically, image ${𝕀}_{i}$ can be represented as

$${𝕀}_{i}=\overset{N_{i}}{\underset{k=1}{⋃}}R_{k}^{\left(i\right)},$$ (1)

and for any $k,k^{\prime }\in \{1,2,\ldots ,N_{i}\}$, we have $R_{k}^{\left(i\right)}⋂R_{k^{\prime }}^{\left(i\right)}=\emptyset$ when $k\neq k^{\prime }$. Here $R_{k}^{\left(i\right)}$ is a fixed-size region of ${𝕀}_{i}$ for all *k*.

To simplify training process, we extract the deep feature of $R_{k}^{\left(i\right)}$ denoted by $r_{k}^{\left(i\right)}$($\in {\mathbb{R}}^{m}$) using a pretrained deep neural network. Let 𝒮 represent the space consisted of any finite set of elements in ${\mathbb{R}}^{m}$. The goal of PNF is to learn a mapping ${𝒜}_{\Psi }$ that transforms elements from 𝒮 to a low-dimensional space $𝒵\subset {\mathbb{R}}^{d}$,

$${𝒜}_{\Psi }:\{r_{k}^{\left(i\right)}{\}}_{k=1}^{N_{i}}\mapsto z_{i},\;𝒮\to 𝒵,$$ (2)

where ${𝒜}_{\Psi }$ is characterized by a learnable-parameter set Ψ.

**Linear transformation.** We first transform an input $H_{\text{in}}\in {\mathbb{R}}^{c_{\text{in}}\times p}$ by a linear mapping $W^{p\times c_{\text{out}}}$ for dimensionality reduction, i.e.,

$$H_{\text{out}}=H_{\text{in}}\cdot W,$$ (3)

and hence $H_{\text{out}}\in {\mathbb{R}}^{c_{\text{in}}\times c_{\text{out}}}$. Note that W can be regarded as an ensemble of one-dimensional convolution.

**Multi-head dot-product attention.** We then propose a multi-head attention to aggregate columns of $H_{\text{out}}$. Consider *M* heads attention, we characterize the similarity relationship between columns of $H_{\text{out}}$ by a scaled inner product

$$\alpha_{i}^{m}=\sum_{j=1,j\neq i}^{c_{\text{out}}}\frac{\langle h_{\text{out},i}⊙\theta^{m},h_{\text{out},j}⊙\theta^{m}\rangle }{c_{\text{out}}},$$ (4)

where $h_{\text{out},\cdot }$ stands for the columns of $H_{\text{out}}$ and $\theta^{m}\in {\mathbb{R}}^{c_{\text{in}}}$. The softmax operator follows for normalization

$${\hat{\alpha }}_{i}^{m}=\exp(\alpha_{i}^{m})/\sum_{j=1}^{c_{\text{out}}}\exp(\alpha_{j}^{m}).$$ (5)

We attain linear combination of element-wise scaled columns as

$$h_{a}^{m}=\sum_{i=1}^{c_{\text{out}}}{\hat{\alpha }}_{i}^{m}(h_{\text{out},i}⊙\theta ).$$ (6)

where $\theta \in {\mathbb{R}}^{c_{\text{in}}}$. By varying *m* from 1 to *M*, we have a multi-head attention representation $H_{a}=[h_{a}^{1},h_{a}^{2},\ldots ,h_{a}^{M}]\in {\mathbb{R}}^{c_{\text{in}}\times M}$.

**Building blocks of PFN.** Let $C_{W}$ and $A_{\Theta }$ denote the linear transformation and the multi-head dot-product attention, respectively. The building block of PFN can be represented as a composition operator $FB=A_{\Theta }\circ \text{Tanh}\circ C_{W}$, where *W* and Θ are learnable parameters in the linear transformation and the multi-head dot-product attention, respectively.

By stacking multiple building blocks, we express PFN ${𝒜}_{\Psi }$ as

$${𝒜}_{\Psi }=FB^{L}\circ (FB^{L-1}{)}^{T}\circ \cdots \circ FB^{1}.$$ (7)

where $(\cdot {)}^{T}$ represents transposition. For the final block, we flatten its output in row-major order, and achieve a low-dimensional representation, denoted by h.

To accommodate with various number of patches of whole-slide images, we perform uniformly sampling with replacement on deep features of patches with a fixed sample size for each image and use the random samples as inputs of PFN. Hence the input of PFN is represented as $H^{i}=[c_{1}^{i},c_{2}^{i},\ldots ,c_{N}^{i}{]}^{T}\in {\mathbb{R}}^{N\times m}$ for the image ${𝕀}_{i}$, where $c_{\cdot }^{i}$ represents a random sample from $\{r_{k}^{\left(i\right)}{\}}_{k=1}^{N_{i}}$ and *N* is the sample size.

**Attention score.** We define attention score of $c_{\cdot }^{i}$ by the output of the first building block.

Let


$${\tilde{H}}^{i}=FB^{1}{\left(H^{i}\right)}^{T}\in {\mathbb{R}}^{M_{1}\times N},$$

where *M*_1_ denotes the number of heads of attention using in the *FB*^1^. The attention score of $c_{k}^{i}$ is defined as

$$\alpha_{k}^{i}=\frac{\exp\left(\sum_{r=1}^{M_{1}}|{\tilde{H}}_{r,k}^{i}|\right)}{\sum_{j=1}^{N}\exp\left(\sum_{r=1}^{M_{1}}|{\tilde{H}}_{r,j}^{i}|\right)},\;k\in \{1,2,\ldots ,N\}.$$ (8)

**Memory cost of dot-product attention.** Given the input $H\in {\mathbb{R}}^{K\times D}$, the memory cost of single-head dot-product attention is *O*(*K*^2^ + *KD*), where the *K*^2^ term arises from computing pairwise inner products between scaled embeddings, and the *KD* term accounts for storage of the scaled embeddings. If *K*<*D*, the quadratic term *K*^2^ can be omitted, reducing the memory cost to *O*(*KD*). Under this condition, dot-product attention can be more memory-efficient than ABMIL and comparable to LongNet. A summary of the comparison with ABMIL and LongNet is provided in Table C in S1 Appendix.

### Masked transcriptomic autoencoder (MTA)

To learn high quality compact representations of transcriptomic data, we propose a modified masked autoencoder, which is implemented by multi-layer perceptrons (MLP). Let $g_{i}\in {\mathbb{R}}^{p}$ denote the transcriptomic data of patient *i* corresponding to the whole-slide image ${𝕀}_{i}$. We randomly mask elements of $g_{i}$ and reconstruct the unmasked elements by a MLP-based autoencoder.

Let $m_{i}\in {\mathbb{R}}^{p}$ be a random vector, where each of its elements is generated by a Bernoulli distribution Bernoulli(ξ). The masked $g_{i}$ is written as

$${\hat{g}}_{i}=g_{i}⊙(1-m_{i}),$$ (9)

The transcriptomic data is reconstructed by a MLP,

$${\tilde{g}}_{i}=\text{MLP}({\hat{g}}_{i}),$$ (10)

where the *l*-th layer of MLP is implemented as

$$z_{i}^{\left(l\right)}=\text{Tanh}(W^{\left(l\right)}z_{i}^{\left(l-1\right)}+b^{\left(l\right)}).$$ (11)

Tanh(·) is employed for its symmetric and bounded characteristics, which avoid activation explosion and promote stable optimization. For positively skewed inputs, the preceding linear projections can adaptively rescale their magnitudes during training, thereby reducing the risk of saturation.

In Eq (11), $W^{\left(l\right)}\in {\mathbb{R}}^{p_{l}\times p_{l-1}}$ and $b^{\left(l\right)}\in {\mathbb{R}}^{p_{l}}$ are learnable parameters and we denote the dimension of the *l*-th layer by *p*_*l*_. Note that $z_{i}^{\left(0\right)}={\hat{g}}_{i}$.

The reconstruction loss of masked transcriptomic data is defined by the squared loss

$$\ell_{recon}=\|\tilde{g}⊙m-g⊙m{\|}_{2}^{2},$$ (12)

where we have omitted the subscript for brevity. The reconstruction loss varies depending on the random mask. Hence each randomly masked version of a patient’s transcriptomic profile can be regarded as an independent sample for data reconstruction.

Given a mini-batch of $ℬ=\{(g_{i},m_{i}){\}}_{i=1}^{B}$ with size *B*, the reconstruction loss is defined by the mean squared error (MSE)

$${ℒ}_{recon}=\frac{1}{B}\sum_{i=1}^{B}\|{\tilde{g}}_{i}⊙m_{i}-g_{i}⊙m_{i}{\|}_{2}^{2}.$$ (13)

MTA learns implicit connections between genes via masked reconstruction. With the random vector $m_{i}$, we are able to generate different versions of masked transcriptomic expression signals for patient *i*. Hence, we also adopt InfoNCE loss [41,42] in MTA. For simplicity, let z be the output of the middle layer for MLP as the latent representation of masked transcriptomic data $\hat{g}$. We define a projection head as

$$\tilde{z}=W_{p}^{\left(2\right)}\text{Tanh}(W_{p}^{\left(1\right)}z+b_{p}^{\left(1\right)})+b_{p}^{\left(2\right)}$$ (14)

Shapes of the linear transformation matrices $W_{p}^{\left(i\right)}$ and biases $b_{p}^{\left(i\right)}$ (i=1,2) are predefined.

Given a mini-batch of projected latent representations $ℬ=\{{\tilde{z}}_{k}{\}}_{k=1}^{B}$with size as *B*, let ${ℬ}_{-k}=ℬ\backslash \{{\tilde{z}}_{k}\}$ and ${𝒫}_{k}$ be the collections of projected latent representations generated from the same patient as ${\tilde{z}}_{k}$. Let $\hat{z}=\tilde{z}/\|\tilde{z}{\|}_{2}$, where subscripts are omitted for brevity. The InfoNCE loss is defined as

$${ℒ}_{con}=\sum_{{\tilde{z}}_{k}\in ℬ}\frac{-1}{\left|{𝒫}_{k}\right|}\sum_{{\tilde{z}}_{p}\in {𝒫}_{k}}\log\frac{\exp({\hat{z}}_{k}^{T}{\hat{z}}_{p}/\tau )}{\sum_{{\tilde{z}}_{a}\in {ℬ}_{-k}}\exp({\hat{z}}_{k}^{T}{\hat{z}}_{a}/\tau )},$$ (15)

where *τ* is the temperature.

### Heterogenous data alignment

**Alignment by orthogonal decomposition.** Given the low-dimensional representation h of a whole-slide image and the corresponding latent representation of transcriptomic data z, both of which are originated from the same patient, we assume the orthogonal decomposition

$$z=h+e\;\text{and}\;h^{T}e=0,$$ (16)

where e denotes the irrelevant part to h.

A straightforward way to align h and z is to minimize $\|h-z{\|}_{2}^{2}$. But it may lead to a trivial solution e=0. Instead, we relax the orthogonal decomposition as the loss function

$$\ell_{algin}=|h^{T}(h-z)|+\frac{\delta }{2}(\|z-h{\|}_{2}^{2}-C{)}^{2}.$$ (17)

where *δ* and *C* are hyper-parameters. Empirically, *δ* is set to balance the scales of each term and *C* is set to 1.

By minimizing $\ell_{algin}$, the first term decomposes the irrelevant part to h from z and the second term achieves alignment between the whole-slide image and the transcriptomic data while avoiding a trivial solution that leads to exact alignment.

**Alignment by partial reconstruction.** We can also establish implicit alignment between h and z by using latent representations of images for transcriptomic reconstruction. Specifically, we transform the output of the middle layer of MTA by a bilinear operator as

$$\dot{z}=(W_{G}z+b_{G})⊙(W_{I}h+b_{I}),$$ (18)

where ⊙ indicates element-wise product. Shapes of $W_{\cdot }$ and $b_{\cdot }$ in Eq (18) depend on the dimensions of z and ***h***. Then $\dot{z}$ passes the next layer in MTA for reconstruction of masked transcriptomic data.

### Data augmentation

In our study, each patient is provide with a whole-slide image and transcriptomic data at the training stage. Denote ${\left\{\left(\{r_{k}^{\left(i\right)}{\}}_{k=1}^{N_{i}},g_{i},y_{i}\right)\right\}}_{i=1}^{M}$ as the training set, where *y*_*i*_ indicates the molecular subtype of patient *i*. We sample with replacement from $\{r_{k}^{\left(i\right)}{\}}_{k=1}^{N_{i}}$ with a fixed sample size *N* and get $H^{i}=[c_{1}^{i},c_{2}^{i},\ldots ,c_{N}^{i}{]}^{T}\in {\mathbb{R}}^{N\times m}$ where $c_{\cdot }^{i}$ represents the random samples. Simultaneously, we generate a random mask $m_{i}$ for the transcriptomic data $g_{i}$. We repeat the procedure multiple times for one sample in the training set. Eventually, we obtain an extended training set denoted by ${\left\{(H^{i},g_{i},m_{i},y_{i})\right\}}_{i=1}^{M^{\prime }}$. We have abused using the index *i* here, but the meaning of *i* should be clear from the context. Note that in the tuple $\left(H^{i},g_{i},m_{i},y_{i}\right)$, the transcriptomic data $g_{i}$ and the molecular subtype *y*_*i*_ are copied from the patient that generates $H^{i}$ and $m_{i}$.

### Total loss

Given a mini-batch of augmented data $ℬ={\left\{(H^{i},g_{i},m_{i},y_{i})\right\}}_{i=1}^{B}$. The latent representation $h_{i}={𝒜}_{\Psi }(H^{i})$ and the ground-truth subtype $y_{i}\in \{1,2,\ldots ,K\}$ are used to construct the cross-entropy loss for training. A linear classifier generates the predicted subtype ${\hat{y}}_{i}$ $\in {\mathbb{R}}^{K}$, i.e.,

$${\hat{y}}_{i}=\text{softmax}(W_{c}h_{i}+b_{c}).$$ (19)

The symbol *K* indicates the number of subtypes. The cross-entropy loss of the batch ℬ is

$${ℒ}_{CE}=\frac{1}{B}\sum_{i=1}^{B}-\log{\hat{y}}_{i,y_{i}}.$$ (20)

By Eq (17), the alignment loss of whole-slide images and transcriptomic data is

$${ℒ}_{align}=\frac{1}{B}\sum_{i=1}^{B}\left(|h_{i}^{T}(h_{i}-z_{i})|+\frac{\delta }{2}(\|z_{i}-h_{i}{\|}_{2}^{2}-C{)}^{2}\right).$$ (21)

With different combinations of loss functions, we get three variants of our method.

- Combing Eqs (13), (15), (20), and (21) yields the total loss
  $${ℒ}_{AOD}={ℒ}_{CE}+\lambda {ℒ}_{align}+{ℒ}_{con}+{ℒ}_{recon}.$$ (TEMI w/AOD)
- Combing Eqs (13), (15), (18), and (20) yields the total loss
  $${ℒ}_{APR}={ℒ}_{CE}+\lambda {ℒ}_{con}+{ℒ}_{recon}.$$ (TEMI w/APR)
- Combing Eqs (13), (15), (18), (20), and (21) yields the total loss
  ${ℒ}_{AOD+APR}={ℒ}_{CE}+\lambda \left({ℒ}_{align}+{ℒ}_{con}\right)+{ℒ}_{recon}.$ (TEMI w/AOD+APR)

The hyperparameter *λ* is set for balancing the scales of individual terms. All learnable parameters are learned by minimizing the total loss.

### Datasets and preprocessing

Three datasets were used to classify microsatellite instable (MSI) and microsatellite stable (MSS) subtypes, with MSI arising from deficiencies in the DNA mismatch repair (MMR) system:

- **CRC-DX:** 358 colorectal cancer (CRC) patients with diagnostic slides (indicated by the *DX* suffix) and bulk transcriptomic profiles retrieved from TCGA colon and rectal adenocarcinomas.
- **CRC-KR:** The same patients as CRC-DX, with *cr*yosections generated from snap-frozen tissues, denoted by the suffix *KR.*
- **STAD-DX:** 284 stomach adenocarcinoma (STAD) patients with diagnostic slides and bulk transcriptomic profiles obtained from TCGA.

Additionally, one dataset was used to classify Proneural (Pron.) and Mesenchymal (Mese.) subtypes of glioblastoma multiforme (GBM), two highly distinct transcriptomic subtypes characterized by differential gene expression [48]:

- **GBM-DX:** 182 GBM patients with diagnostic images and bulk transcriptomic profiles retrieved from TCGA.

All datasets used HTSeq-FPKM records as bulk transcriptmoic profiles. For while-slide images, CRC-DX, STAD-DX, and GBM-DX utilized diagnostic slides from formalin-fixed paraffin-embedded tissues, while CRC-KR used cryosections from snap-frozen tissues.

The three datasets for MSI vs. MSS classification were sourced from [8]. The whole-slide images had been tiled into non-overlapped patches with size 224 pixel×224 pixel at a resolution of 0.5μm/pixel and had been conducted color normalization with the Macenko method. The tumor patches were identified by a well-trained ResNet18. We extracted 512-dimensional deep features of tumor patches from the last pooling layer of this pre-trained ResNet18 as inputs of our methods.

In GBM-DX, the diagnostic slides at 20x magnification were tiled into 512 pixel×512 pixel patches and the non-informative regions were filtered by the Otsu’s method. Each patch was resized as 224 pixel×224 pixel to be compatible with the input size of ResNet18. Notably, a resize-free alternative pipeline is to tile WSIs directly into patches of size 224 pixel×224 pixel and store them in memory-efficient formats (e.g., OME-Zarr), which can be accessed via a PyTorch DataLoader [49]. Patches from different slides but the same patient were merged. The rest procedure followed the same pipeline as the three datasets used for MSI vs. MSS classification.

For HTSeq-FPKM records, genes with more than 20% missing values were excluded, and the top 2000 most variable genes were selected. Subsequently, a log(1+x) transformation was applied to the data.

We fixed the sample size *N* = 200 and repeated 100 times for collections of deep features of patches for one patient to generate the augmented dataset. See the Data augmentation section for details.

The training set and the test set were randomly splitted at patient-level for each cohort. In CRC-DX, 258 patients were used for training and 100 patients were used for test. After data augmentation, there were 25800 samples in the training set and 10000 samples in the test set. In GBM-DX, 127 patients were used for training and 55 patients were used for test. Similarly, we built a training set including 12700 samples and a test set including 5500 samples through data augmentation. There were 185 patients for training and 99 patients for test in STAD-DX. In CRC-KR, 278 patients were used for training and 109 patients were used for test. Through data augmentation, training sets and test sets were 100 times larger than the number of patients in each cancer cohort. A summary of datasets is presented in Table D S1 Appendix.

### Technical details

**Network architectures.** We implemented PFN with two stacked building blocks. We summarized the shapes of parameters of each block in Fig G in S1 Appendix. The backbone of MTA consisted of six linear layers. The dimensional structure of layers is 2000-512-128-32-128-1024-2000. MTA’s projection head was implemented by a two-layer fully connected network with structure 32-64-128. Batch normalization was applied before hidden features went through activation functions. The network architecture in a pytorch-style was presented Fig H in S1 Appendix.

**Label smoothing.** We applied label smoothing [50] in the cross-entropy loss Eq (20). Suppose $y\in \{0,1{\}}^{K}$ is a one-hot coding of label y∈{1,2,…,K} and $\hat{y}\in \Delta_{K}$ is the predicted likelihood that assigns to each class, where $\Delta_{K}$ is the *K*-dimensional simplex. Then the individual loss of the pair $\left(y,\hat{y}\right)$ is rewritten as

$$\sum_{k=1}^{K}{\tilde{y}}_{k}\log{\hat{y}}_{k}$$ (22)

when label smoothing is applied, where ${\tilde{y}}_{k}=y_{k}(1-\alpha )+\alpha /K$ and *α* is a hyperparameter in the range of [0,1].

**Parameter settings.** We trained our model by AdamW with 1e-3 as learning rate. The batch size was 2048. The temperature *τ* in Eq (15) was 0.05. The *δ* in Eq (21) and the *λ* in various total losses were set as 0.1 and 0.5, respectively, for balancing loss scales. The number of epochs varied in different datasets and total losses. It depended on the model’s performance over training sets. In (TEMI w/AOD), the number of epochs when training CRC-DX and STAD-DX was set as 15 and it was 50 in training GBM-DX. In (TEMI w/APR) and (TEMI w/AOD+APR), the numbers of epochs were 15, 25, and 50 for CRC-DX, STAD-DX, and GBM-DX, respectively. We used a larger learning rate 5e-3 when training GBM-DX with (TEMI w/APR) and (TEMI w/AOD+APR). The parameter *α* for label smoothing was set as 0.05, 0.1, and 0.01 for CRC-DX, STAD-DX, GBM-DX, respectively. The masked ratio ξ was 0.7 in MTA. For scenarios that advanced foundation models were used as feature extractors in CRC-DX, the training parameters—including learning rate, optimizer, and numbers of epochs—are summarized in Table E in S1 Appendix.

**Patch-level attention scores**. We computed the final attention score of a patch by averaging its scores obtained from Eq (8) over the 100 repetitions for sampling.

### Evaluation

**Metrics.** We evaluated models using the area under the receiver operating characteristic curve (AUC). The metric was computed at the patient level, with prediction for samples from the same patient aggregated by majority voting.

**Bootstrapping.** To ensure sufficient data for both training and testing, we did not create a separate independent validation set. To address this limitation, we performed 1,000-fold bootstrapping. The median and 95% confidence intervals for AUC are reported.

## Alternative heterogenous data alignment

Data alignment is prevalent in multi-modal learning and transfer learning [51,52]. There are serveral typical criteria for data alignment. Suppose we are given a min-batch of pair-representation from two different data sources ℋ and 𝒵 denoted by $\{(h_{i},z_{i}){\}}_{i=1}^{B}$.

**Mean squared error.** The mean squared error describes an exact alignment between different data sources.

$$ℒ=\frac{1}{B}\sum_{i=1}^{B}\|h_{i}-z_{i}{\|}_{2}^{2}.$$ (23)

**Similarity consistency.** Similarity consistency assumes similarity as an invariant metric in different representation spaces. Define the similarities between data points in ℋ and 𝒵, respectively, as

$$s_{ℋ,ij}=\frac{\exp(h_{i}^{T}h_{j}/\tau )}{\sum_{k=1}^{B}\exp(h_{i}^{T}h_{k}/\tau )},$$ (24)

and

$$s_{𝒵,ij}=\frac{\exp(z_{i}^{T}z_{j}/\tau )}{\sum_{k=1}^{B}\exp(z_{i}^{T}z_{k}/\tau )}.$$ (25)

The similarity consistency is defined by Frobenius norm of the difference between similarity matrices

$$ℒ=\|S_{ℋ}-S_{𝒵}{\|}_{F}.$$ (26)

where $S_{\cdot }=[s_{\cdot ,ij}{]}_{B\times B}$.

**Hilbert-Schmidt independence criterion (HSIC).** HSIC measures independence between two data sources ℋ and 𝒵 in a reproducing kernel Hilbert space [53]. Its empirical estimator is written as

$$\text{HSIC}(ℋ,𝒵)=\frac{1}{(B-1{)}^{2}}\text{trace}(K_{ℋ}\Lambda K_{𝒵}\Lambda ),$$ (27)

where $K_{ℋ}=[k_{ℋ}(h_{i},h_{j}){]}_{B\times B}$ and $K_{𝒵}=[k(z_{i},z_{j}){]}_{B\times B}$, defined by reproducing kernels $k_{ℋ}(\cdot ,\cdot )$ and $k_{𝒵}(\cdot ,\cdot )$, respectively, and $\Lambda =I-11^{T}/B\in {\mathbb{R}}^{B\times B}$. The symbol I indicates a *B*-by-*B* identity matrix, and 1 is a column vector with all its elements as 1. If HSIC(ℋ,𝒵)=0, then data points from two different data sources are independence. Thus, we maximize the criterion to achieve alignment. The loss function is

ℒ=−HSIC(ℋ,𝒵). (28)

**Maximum mean discrepancy (MMD).** MMD is a well-known divergence that measures the distance between distributions [54]. It is commonly used for minimizing the gap between data sources in domain adaptation. Let $P_{ℋ}$ and $P_{𝒵}$ denote distributions. A biased MMD estimator is

$$ℒ={\left[\frac{1}{B^{2}}\sum_{i,j=1}^{B}k(h_{i},h_{j})-\frac{2}{B^{2}}\sum_{i,j=1}^{B}k(h_{i},z_{j})+\frac{1}{B^{2}}\sum_{i,j=1}k(z_{i},z_{j})\right]}^{\frac{1}{2}}.$$ (29)

where k(·,·) is a kernel defined on the support set of $P_{H}$ and $P_{𝒵}$.

**Gaussian Wasserstein distance.** Gaussian Wasserstein distance is a divergence that measures the difference between Gaussian distribution [55]. Given Gaussian distributions $N(m_{H},\Sigma_{H})$ and $N(m_{𝒵},\Sigma_{𝒵})$ on the support set $𝒰\subset {\mathbb{R}}^{d}$, the 2-Wasserstein distance is written as

$$\|m_{ℋ}-m_{𝒵}{\|}_{2}^{2}+\text{trace}\left(\Sigma_{ℋ}+\Sigma_{𝒵}-2{\left(\Sigma_{ℋ}^{\frac{1}{2}}\Sigma_{𝒵}\Sigma_{ℋ}^{\frac{1}{2}}\right)}^{\frac{1}{2}}\right)$$ (30)

From simplicity, we assume that $\Sigma_{H}$ and $\Sigma_{𝒵}$ are commuting. The Eq (30) can be simplified as

$$L=\|m_{H}-m_{𝒵}{\|}_{2}^{2}+\|\Sigma_{H}^{\frac{1}{2}}-\Sigma_{𝒵}^{\frac{1}{2}}{\|}_{F}^{\frac{1}{2}}.$$ (31)

Note that data points from different sources are not necessarily to be paired in computing HSIC, MMD, or 2-Wasserstein distance.

Gaussian kernel with bandwidth $k(h,z)=\exp\left(-\frac{\|h-z{\|}_{2}^{2}}{\delta }\right)$ is commonly used when computing HSIC and MMD. The bandwidth *δ* takes the average of Euclidean distances between data points.

These alignment criteria assume exact alignment between data sources, whether in terms of representation or distribution. In contrast, given the substantial differences between whole-slide images and molecular signals, our approach acknowledges that only partial alignment of information is possible.

## Supporting information

S1 Appendix Supplementary Materials. This supporting document contains all supplementary tables and figures cited in the main text. It includes the following sections:
Enrichment analysis in STAD-DXAnalysis of attention scoresFoundation models as feature extractorsDistributions of mean squared errors in GBM-DXMemory cost of the dot-product attentionDescriptions of datasetsArchitecture of patch fusion networkArchitecture of masked transcriptomic autoencoderTraining settings of TEMI with foundation-model features
(PDF)
