# Supplementary material for: Transcriptomic-guided whole-slide image classification for molecular subtype identification
Source: PLoS Comput Biol. 2026 Feb 9;22(2):e1013950. doi: 10.1371/journal.pcbi.1013950 (PMC12900446; doi:10.1371/journal.pcbi.1013950)
Supplement: S1 Appendix — This supporting document contains all supplementary tables and figures cited in the main text. It includes the following sections: Enrichment analysis in STAD-DXAnalysis of attention scoresFoundation models as feature extractorsDistributions of mean squared errors in GBM-DXMemory cost of the dot-product attentionDescriptions of datasetsArchitecture of patch fusion networkArchitecture of masked transcriptomic autoencoderTraining settings of TEMI with foundation-model features (PDF) [file pcbi.1013950.s001.pdf]

Transcriptomic-guided whole-slide image classification for molecular subtype identification: Supplementary Materials

Weiwen Wang[1*], Xiwen Zhang[2], Yuanyan Xiong[3],
1 Department of Mathematics,
College of Information Science and Technology, Jinan University,
Guangzhou, Guangdong, China
2 Department of Bioinformatics,
College of Medical Information Engineering, Guangdong Pharmaceutical University,
Guangzhou, Guangdong, China
3 Department of Biochemistry,
Key Laboratory of Gene Engineering of the Ministry of Education,
School of Life Sciences, Sun Yat-sen University,
GuangZhou, Guangdong, China
* To whom correspondence should be addressed.
Email: wangww29@jnu.edu.cn

Contents

1 Enrichment analysis in STAD-DX **3**

2 Analysis of attention scores **4**

3 Foundation models as feature extractors **8**

4 Distributions of mean squared errors in GBM-DX **9**

5 Memory cost of the dot-product attention **10**

6 Descriptions of datasets **11**

7 Architecture of patch fusion network **12**

8 Architecture of masked transcriptomic autoencoder **13**

9 Training settings of TEMI with foundation-model features **14**

References **15**

List of legends of supporting figures and tables **16**

1 Enrichment analysis in STAD-DX

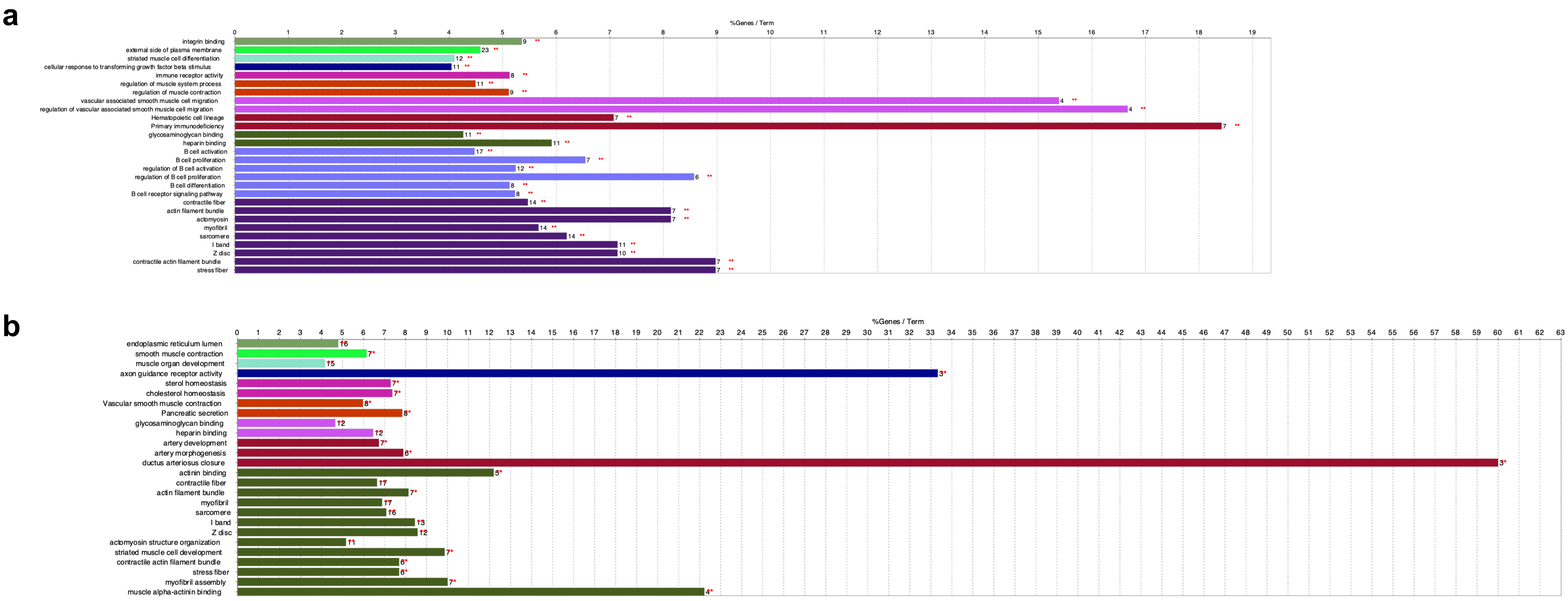

Fig A: **Results of enrichment analysis of top 200 genes with smallest mean squared errors in STAD-DX.** a, Microsatellite stability. b, Microsatellite instability.

2 Analysis of attention scores

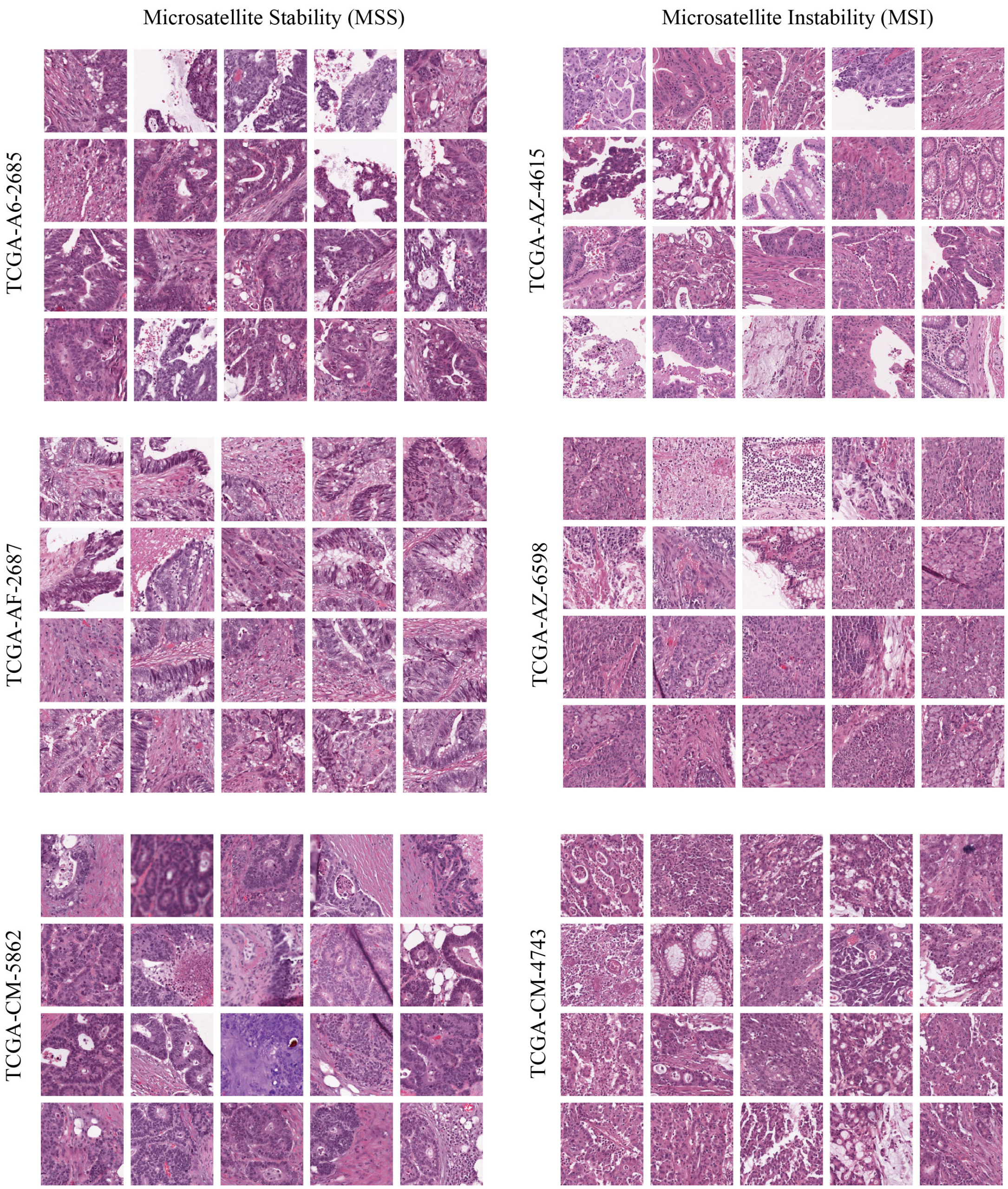

Fig B: **Top 20 patches ranked by attention scores of test samples from CRC-DX.**

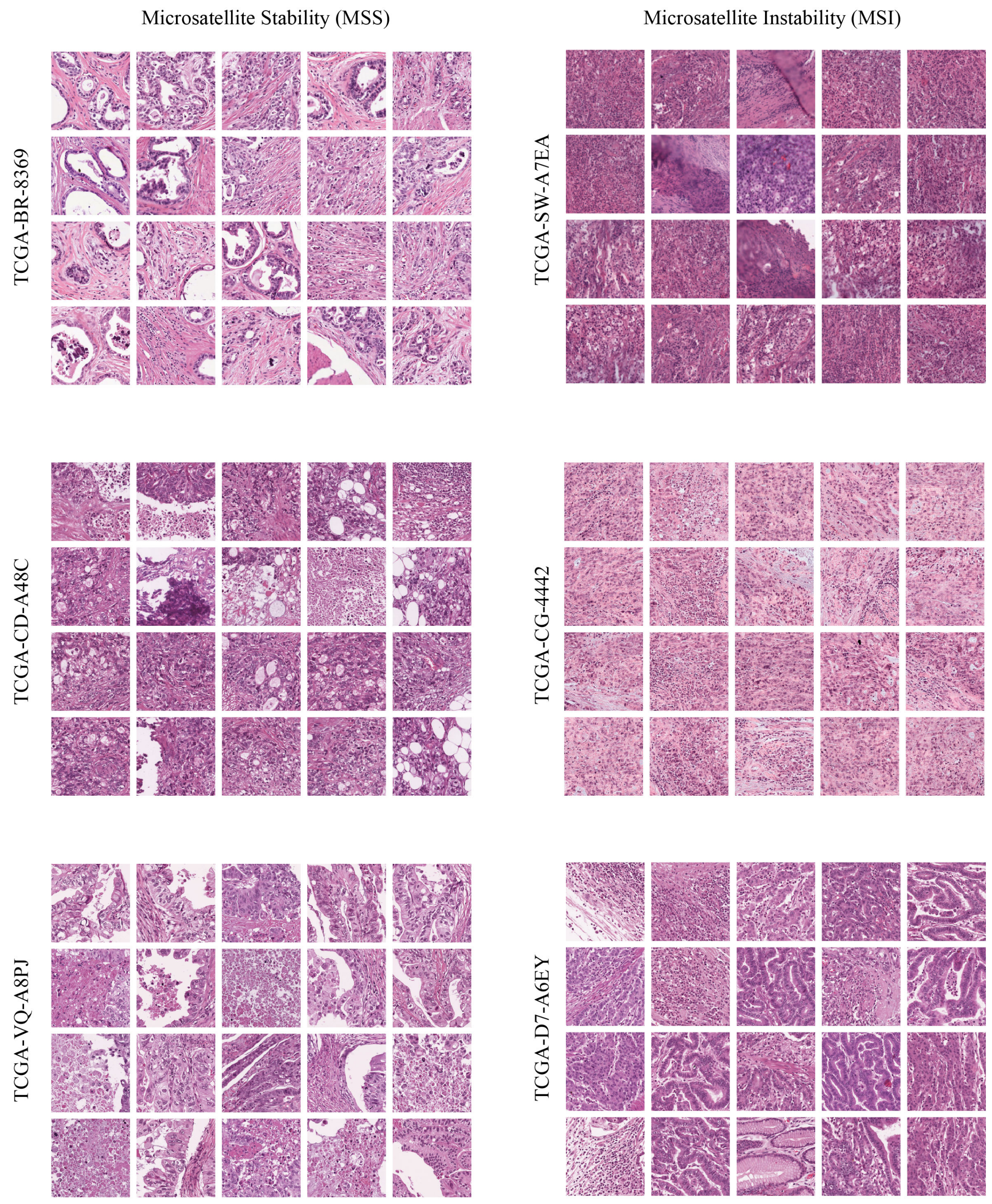

Fig C: **Top 20 patches ranked by attention scores of test samples from STAD-DX.**

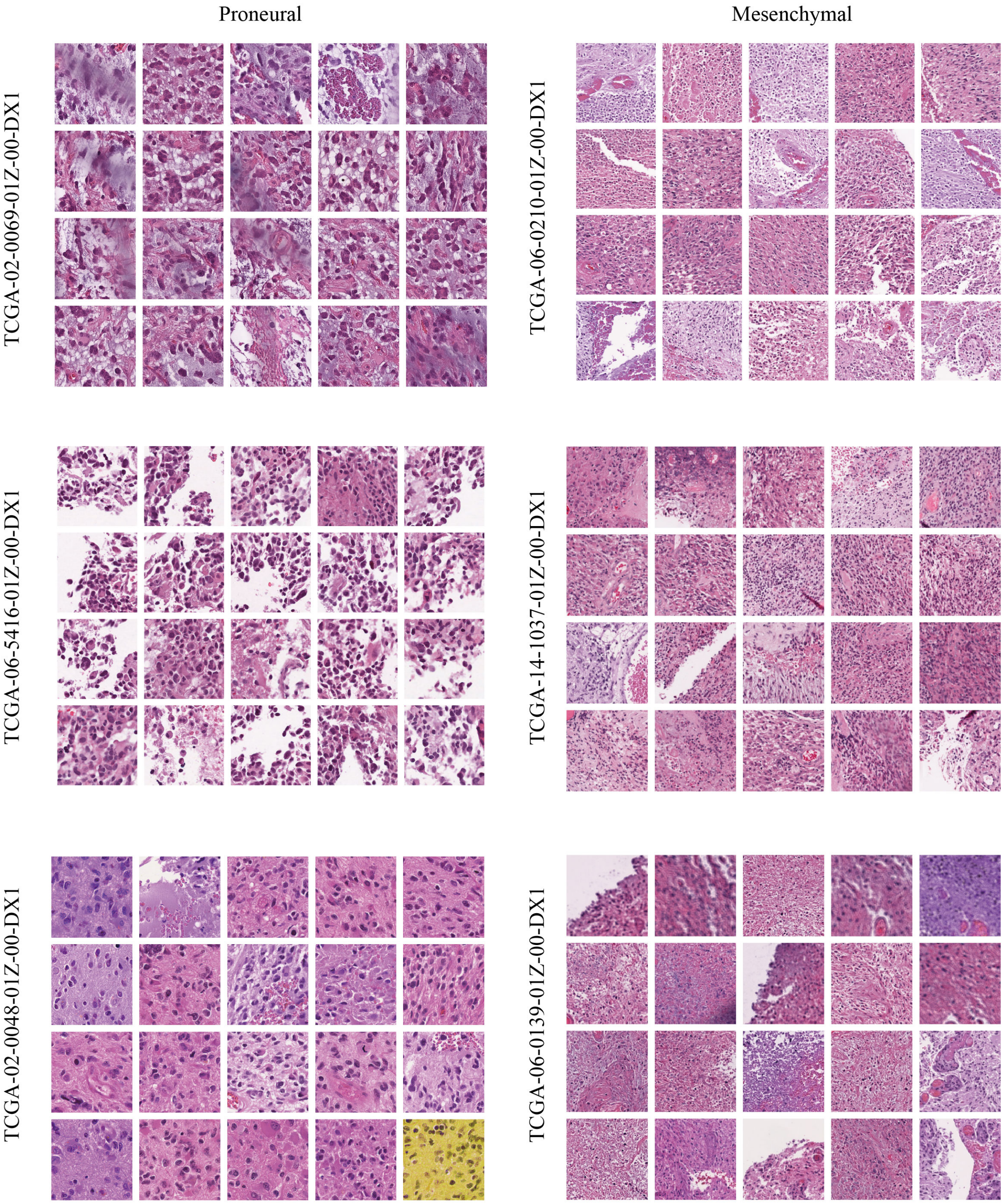

Fig D: **Top 20 patches ranked by attention scores of test samples from GBM-DX.**

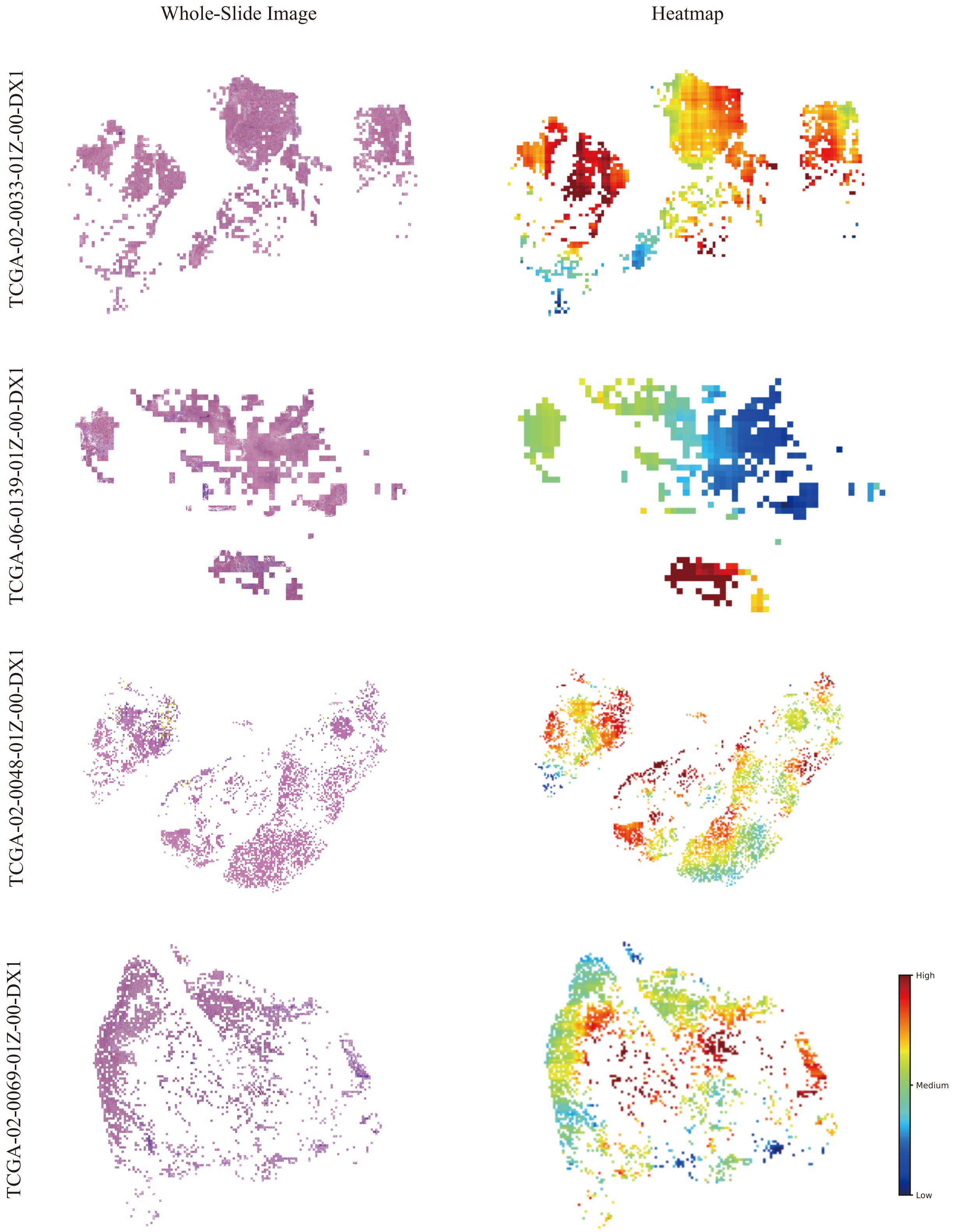

Fig E: **Heatmap of min-max normalized attention scores of patches from four GBM-DX test samples.** Patches of slides are incomplete as only patches identified as tumor regions were used for classification.

3 Foundation models as feature extractors

Table A: **Descriptions of foundation models used as feature extractors.** The dimension of embeddings (Dim. of Embed) determines the input size of TEMI.

**Foundation Model**	**Architecture**	**# Parameter**	**Dim. of Embed.**
DINOv2[3]	ViT-base	86.58M	768
ProvGigaPath[4]	ViT-giant	1134.95M	1536
H-optimus-1[5]	ViT-huge	1134.77M	1536
Virchow[6]	ViT-huge	631.23M	2560

Table B: **Performance of compared methods on the CRC-DX cohort with pathology foundation models as feature extractors.** The feature extractor ResNet18 is used as a baseline. The median and 95% confidence intervals (in parentheses) of AUC from 1000-fold bootstrapping for test samples are reported.

**Methods**	**ResNet18**	**Prov-GigaPath**	**H-optimus-1**	**Virchow**
1Dconv	0.8924 (0.7980-0.9546)	0.9113 (0.8163-0.9726)	0.9095 (0.8118-0.9782)	0.9041 (0.8107-0.9734)
ABMIL	0.8551 (0.7470-0.9386)	0.9057 (0.8176-0.9743)	0.9099 (0.8213-0.9802)	0.9021 (0.8100-0.9660)
TEMI w/o G	0.9043 (0.8259-0.9608)	0.9127 (0.8349-0.9746)	0.9150 (0.8331-0.9779)	0.9116 (0.8167-0.9727)
1Dconv w/ G	0.8944 (0.7971-0.9643)	0.9114 (0.8217-0.9757)	0.9134 (0.8238-0.9797)	0.9158 (0.8274-0.9744)
ABMIL w/ G	0.8862 (0.7833-0.9582)	0.9163 (0.8264-0.9792)	0.9146 (0.8159-0.9806)	0.9071 (0.8141-0.9724)
TEMI w/ AOD	0.9148 (0.8377-0.9661)	0.9229 (0.8446-0.9756)	**0.9214** (0.8402-0.9751)	**0.9132** (0.8368-0.9707)
TEMI w/ APR	0.9116 (0.8412-0.9634)	0.9210 (0.8579-0.9691)	0.9137 (0.8384-0.9688)	0.9117 (0.8205-0.9705)
TMEI w/ AOD+APR	**0.9232** (0.8624-0.9720)	**0.9313** (0.8716-0.9784)	0.9200 (0.8408-0.9776)	**0.9132** (0.8325-0.9666)

4 Distributions of mean squared errors in GBM-DX

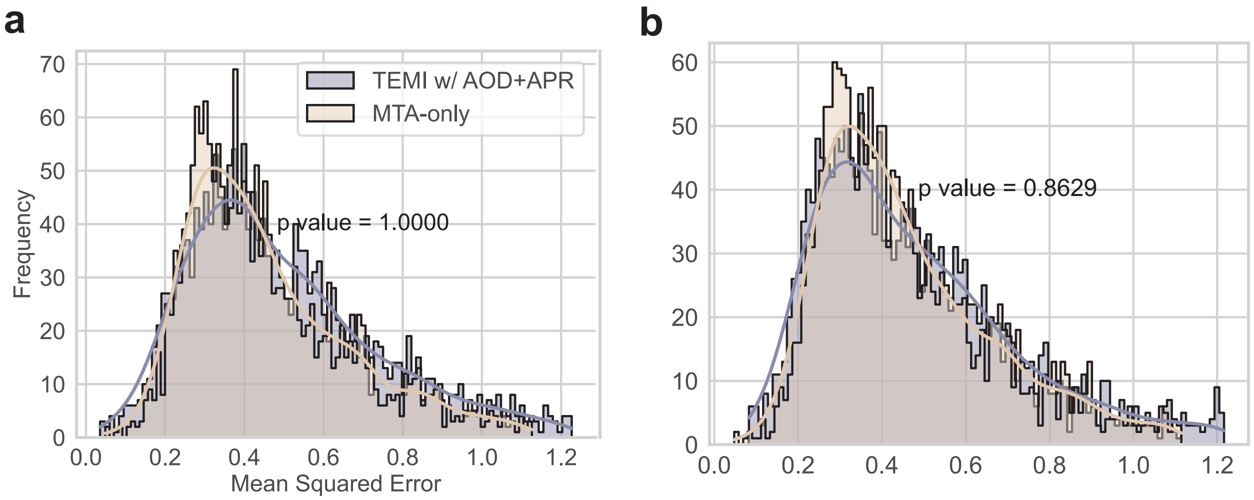

Fig F: **Distributions of mean squared errors for gene expression reconstruction on GBM-DX test samples.** a, The Proneural subtype. b, The Mesenchymal subtype. One-sided Mann-Whitney U-test was conducted to determine whether MSEs of TMEI w/ AOD+APR were significantly lower than that of MTA. P values are annotated in the figures.

5 Memory cost of the dot-product attention

Table C: **Memory complexity comparison of different attention methods.** Given K embeddings $\{\boldsymbol{h}_1, \boldsymbol{h}_2, \ldots, \boldsymbol{h}_K\} \in \mathbb{R}^D$ and the linear projection $\boldsymbol{V} \in \mathbb{R}^{D' \times D}$ in ABMIL, the forward memory costs of the compared methods are reported. Note that if $K < D$, the memory cost of the dot-product attention and LongNet can be reduced to $O(KD)$, whereas ABMIL includes an additional term DD' that accounts for the linear projection.

**Method**	**Memory Complexity (Forward)**
Dot-product Attention	$O(K^2 + KD)$
ABMIL[1]	$O(KD + KD' + DD')$
LongNet[2]	$O(K \log K + KD)$

6 Descriptions of datasets

Table D: **Summary of datasets**. In our experiments, we only used test set of CRC-KR for validation of transfer learning.

Dataset		#MSI	#MSS	#Total
CRC-DX	Train	38	220	258
	Test	26	74	100
CRC-KR	Train	–	–	–
	Test	30	79	109
STAD-DX	Train	35	150	185
	Test	25	74	99
Dataset		#Proneural	#Mesenchymal	#Total
GBM-DX	Train	61	66	127
	Test	27	28	55

7 Architecture of patch fusion network

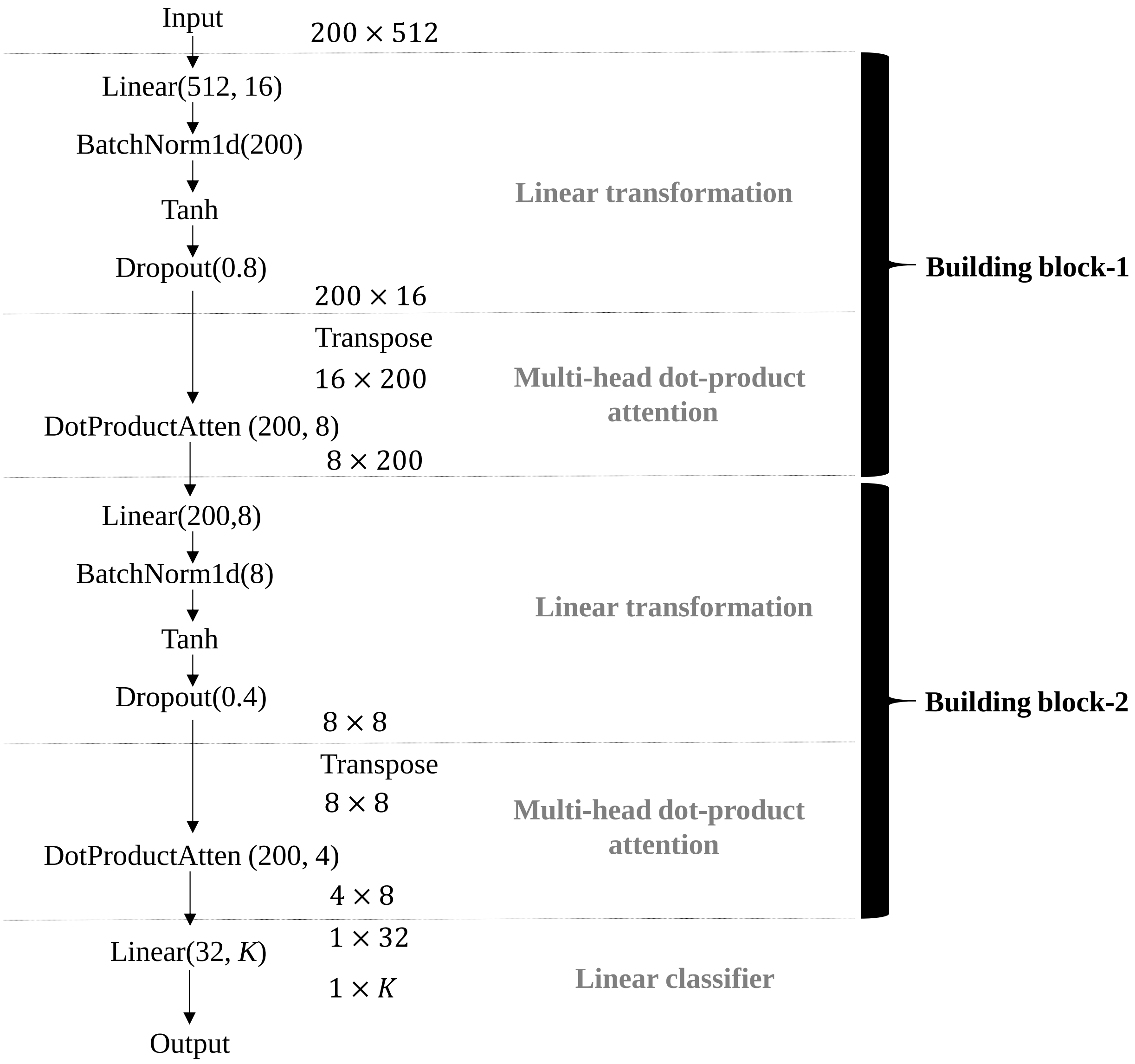

Fig G: **Architecture of patch fusion network in a pytorch-style.** The first and the second parameters in DotProductAtt$(\cdot, \cdot)$ specify the dimension and the number of heads, respectively, for multi-head dot-product attention. We flatten the output of the second building block in row-major. The number K depends on the number of classes. For storage convenience, a transpose operator is applied before multi-head dot-product attention, which is different from what we describe in the main text that the transpose operator is applied between building blocks. Both lead to the same result.

8 Architecture of masked transcriptomic autoencoder

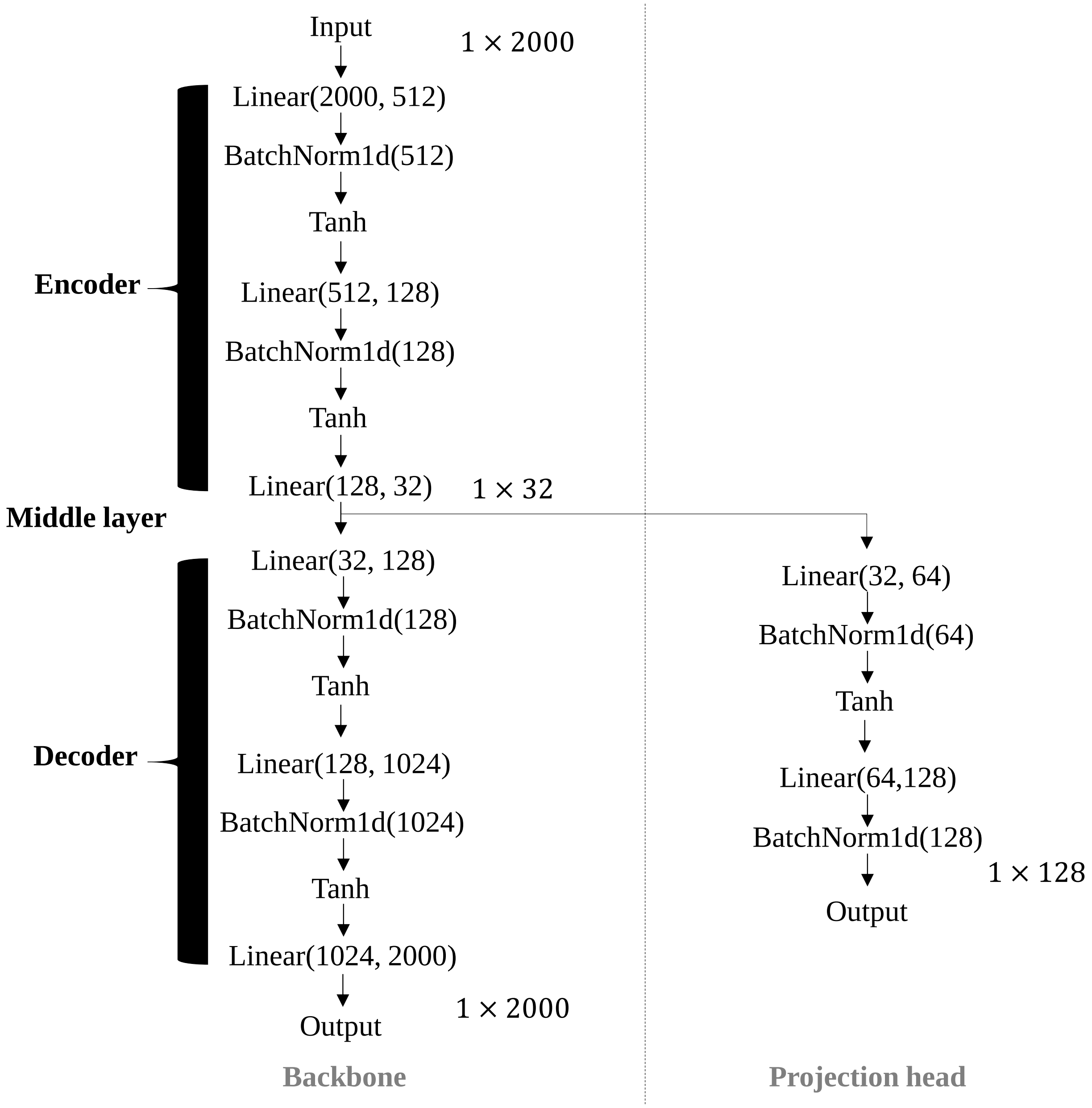

Fig H: **Architecture of masked transcriptomic autoencoder a pytorch-style.** The middle layer is used for data alignment and self-supervised learning.

9 Training settings of TEMI with foundation-model features

Table E: **The training parameters of variants of TEMI when using foundation models as feature extractors.** For all cases, the batch size is set as 2048.

Models	Foundation Models	Learning Rate	# Epoch	Optimizer
TEMI w/o G	DINOv2-base	–	–	–
	Prov-GigaPath	1.00×10^{-3}	1	AdamW
	H-optimus-1	1.00×10^{-3}	1	
	Vrichow	1.00×10^{-3}	1	
TEMI w/ AOD	DINOv2-base	7.00×10^{-4}	20	RMSprop
	Prov-GigaPath	1.00×10^{-3}	1	
	H-optimus-1	1.00×10^{-3}	1	
	Vrichow	1.00×10^{-3}	1	
TEMI w/ APR	DINOv2-base	7.00×10^{-4}	30	RMSprop
	Prov-GigaPath	1.00×10^{-4}	2	
	H-optimus-1	1.00×10^{-4}	2	
	Vrichow	1.00×10^{-3}	2	
TEMI w/ AOD+APR	DINOv2-base	7.00×10^{-4}	20	RMSprop
	Prov-GigaPath	1.00×10^{-3}	5	
	H-optimus-1	7.00×10^{-4}	5	
	Vrichow	1.00×10^{-3}	5	

References

[1] Ilse, M., Tomczak, J. & Welling, M. Attention-based deep multiple instance learning In: *Proceedings of the 35th International Conference on Machine Learning (ICML 2018)* 2127-2136 (2018).

[2] Ding, J., Ma, S., Dong, L., Zhang, X., Huang, S., Wang, W., & Wei, F. Longnet: Scaling transformers to 1,000,000,000 tokens. Preprint at https:// doi.org/10.48550/arXiv.2307.02486 (2023).

[3] Oquab, M., Darcet, T., Moutakanni, T., Vo, H., Szafraniec, M., Khalidov, V., Fernandez, P., Haziza, D., Massa, F., El-Nouby, A. & Assran, M. Dinov2: Learning robust visual features without supervision. Preprint at https://arxiv.org/abs/2304.07193 (2023).

[4] Xu, H., Usuyama, N., Bagga, J., Zhang, S., Rao, R., Naumann, T., Wong, C., Gero, Z., González, J., Gu, Y., Xu, Y., Wei, M., Wang, W., Ma, S., Wei, F., Yang, J., Li, C., Gao, J., Rosemon, J., Bower, T., Lee, S., Weerasinghe, R., Wright, B.J., Robicsek, A., Piening, B., Bifulco, C., Wang, S. & Poon, H. A whole-slide foundation model for digital pathology from real-world data *Nature* **630**, 181-188 (2024).

[5] Bioptimus. H-optimus-1. https://huggingface.co/bioptimus/H-optimus-1 (2025).

[6] Vorontsov, E., Bozkurt, A., Casson, A., Shaikovski, G., Zelechowski, M., Severson, K., Zimmermann, E., Hall, J., Tenenholtz, N., Fusi, N., Yang, E., Mathieu, P., van Eck, A., Lee, D., Viret, J., Robert, E., Wang, Y.K., Kunz, J.D., Lee, M. C. H., Bernhard, J.H., Godrich, R.A., Oakley, G., Millar, E., Hanna, M., Wen, H., Retamero, J.A., Moye, W.A., Yousfi, R., Kanan, C., Klimstra, D.S., Rothrock, B., Liu, S., & Fuchs, T.J. A foundation model for clinical-grade computational pathology and rare cancers detection *Nat. Med.* **30**, 2924–2935 (2024).

List of legends of supporting figures and tables

- **Fig A:** Results of enrichment analysis of top 200 genes with smallest mean squared errors in STAD-DX. a, Microsatellite stability. b, Microsatellite instability.
- **Fig B:** Top 20 patches ranked by attention scores of test samples from CRC-DX.
- **Fig C:** Top 20 patches ranked by attention scores of test samples from STAD-DX.
- **Fig D:** Top 20 patches ranked by attention scores of test samples from GBM-DX.
- **Fig E:** Heatmap of min-max normalized attention scores of patches from four GBM-DX test samples. Patches of slides are incomplete as only patches identified as tumor regions were used for classification.
- **Table A:** Descriptions of foundation models used as feature extractors. The dimension of embeddings (Dim. of Embed) determines the input size of TEMI.
- **Table B:** Performance of compared methods on the CRC-DX cohort with pathology foundation models as feature extractors. The feature extractor ResNet18 is used as a baseline. The median and 95% confidence intervals (in parentheses) of AUC from 1000-fold bootstrapping for test samples are reported.
- **Fig F:** Distributions of mean squared errors for gene expression reconstruction on GBM-DX test samples. a, The Proneural subtype. b, The Mesenchymal subtype. One-sided Mann-Whitney U-test was conducted to determine whether MSEs of TMEI w/ AOD+APR were significantly lower than that of MTA. P values are annotated in the figures.
- **Table C:** Memory complexity comparison of different attention methods. Given K embeddings $\{\boldsymbol{h}_1, \boldsymbol{h}_2, \ldots, \boldsymbol{h}_K\} \in \mathbb{R}^D$ and the linear projection $\boldsymbol{V} \in \mathbb{R}^{D' \times D}$ in ABMIL, the forward memory costs of the compared methods are reported. Note that if $K < D$, the memory cost of the dot-product attention and LongNet can be reduced to $O(KD)$, whereas ABMIL includes an additional term DD' that accounts for the linear projection.
- **Table D:** Summary of datasets. In our experiments, we only used test set of CRC-KR for validation of transfer learning.
- **Fig G:** Architecture of patch fusion network in a pytorch-style. The first and the second parameters in DotProductAtt$(\cdot, \cdot)$ specify the dimension and the number of heads, respectively, for multi-head dot-product attention. We flatten the output of the second building block in row-major. The number K depends on the number of classes. For storage convenience, a transpose operator is applied before multi-head dot-product attention, which is different from what we describe in the main text that the transpose operator is applied between building blocks. Both lead to the same result.

- **Fig H:** Architecture of masked transcriptomic autoencoder a pytorch-style. The middle layer is used for data alignment and self-supervised learning.
- **Table E:** The training parameters of variants of TEMI when using foundation models as feature extractors. For all cases, the batch size is set as 2048.
